# Temporal differentiation of bovine airway epithelial cells grown at an air-liquid interface

**DOI:** 10.1038/s41598-018-33180-w

**Published:** 2018-10-05

**Authors:** Daniel Cozens, Erin Sutherland, Francesco Marchesi, Geraldine Taylor, Catherine C. Berry, Robert L. Davies

**Affiliations:** 1 Institute of Infection, Immunity and Inflammation, College of Medical, Veterinary and Life Sciences, University of Glasgow, Glasgow, UK; 2 School of Veterinary Medicine, University of Glasgow, Glasgow, UK; 3 The Pirbright Institute, Pirbright, Surrey, UK; 4 Institute of Molecular, Cell and Systems Biology, College of Medical, Veterinary and Life Sciences, University of Glasgow, Glasgow, UK

## Abstract

There is an urgent need to develop improved, physiologically-relevant *in vitro* models of airway epithelia with which to better understand the pathological processes associated with infection, allergies and toxicological insults of the respiratory tract of both humans and domesticated animals. In the present study, we have characterised the proliferation and differentiation of primary bovine bronchial epithelial cells (BBECs) grown at an air-liquid interface (ALI) at three-day intervals over a period of 42 days from the introduction of the ALI. The differentiated BBEC model was highly representative of the *ex vivo* epithelium from which the epithelial cells were derived; a columnar, pseudostratified epithelium that was highly reflective of native airway epithelium was formed which comprised ciliated, goblet and basal cells. The hallmark defences of the respiratory tract, namely barrier function and mucociliary clearance, were present, thus demonstrating that the model is an excellent mimic of bovine respiratory epithelium. The epithelium was fully differentiated by day 21 post-ALI and, crucially, remained healthy and stable for a further 21 days. Thus, the differentiated BBEC model has a three-week window which will allow wide-ranging and long-term experiments to be performed in the fields of infection, toxicology or general airway physiology.

## Introduction

The respiratory tract is constantly exposed to a wide variety of potentially harmful matter, including microbes, allergens and particulate material, during the process of inhalation. The airway epithelium represents the first point of contact for inhaled substances and, as such, plays a critical role in protecting the lungs from environmental insults and in maintaining homeostasis^1–4^. The respiratory epithelium provides a physicochemical barrier against inhaled microorganisms and particulates which involve the presence of intercellular junctions^3,5^ and mucociliary clearance^6–8^. However, the barrier function of the respiratory epithelium, together with associated innate immune defences^1,9,10^, can be disrupted by pathogens and this may lead to extensive epithelial damage and transmigration of pathogens to deeper tissue^11,12^. Following injury, the airway epithelium is capable of repair through the proliferation and differentiation of progenitor basal cells and, in this way, the integrity of the respiratory tract is maintained^13–15^. Due to the impact of respiratory pathologies on human and animal health, and the economic and ethical implications associated with animal experimentation, there is an urgent need to develop improved, physiologically-relevant *in vitro* models of the airway epithelium which can be used to better understand the above processes.

Differentiated airway epithelial cells (AECs) are being increasingly used as an *in vitro* tool in both toxicology^16–18^ and infectious disease^19–31^ research involving the respiratory tract. The differentiation of AECs from primary cells is triggered by exposure to an air-liquid interface (ALI) and to specific growth factors and hormones within the culture medium^32–37^. Such differentiated epithelia not only comprise the major cell types (including ciliated, goblet and basal cells) that are associated with the native tissue but also possess its typical pseudostratified architecture^37–40^. The process of epithelial cell differentiation occurs through a number of step-wise stages involving cell proliferation, spreading and migration, cytoskeletal reorganisation and secretion of extracellular matrix^15,27,39^. Importantly, differentiated AEC cultures possess both mucociliary clearance and barrier functions^27,34,39^, characteristics which are critical for assessing the response of the epithelium to challenge with both pathogens^25,27,41,42^ and pollutants^43–45^. Furthermore, since differentiated AEC cultures comprise a mixed population of cell types, they allow the identification of the individual cell-types that are targeted by bacterial^19,23,27^ and viral^20,22,30,31,46^ pathogens. Thus, differentiated AECs provide excellent *in vitro* tools for researching respiratory pathologies.

Differentiated bovine AECs have previously been used to study not only the physiology of the mammalian respiratory tract^47–50^ but also, more specifically, to investigate the pathogenesis of economically-important bacterial and viral pathogens of cattle^28,46,51^. The benefit of using primary cells isolated from abattoir-slaughtered cattle, compared to human tissue, is their ready availability and low cost^47^. Thus, bovine AECs derived from abattoir material represent a more accessible alternative to human cells that are also relevant to the One Health approach of studying infectious disease. Bovine and human respiratory syncytial viruses (RSV), for example, are closely related viruses that cause similar infections in cattle and humans, respectively; indeed, a bovine RSV animal model has been employed to study the pathogenesis of, and develop improved therapeutics against, human RSV infection^52–54^. Thus, due to the greater ease and lower cost of acquiring primary bovine airway epithelial cells, a bovine RSV infection model utilising differentiated bovine AECs could be used to model human RSV pathogenesis *in vitro*.

To accurately assess respiratory tract pathologies following exposure to infection or pollutants, differentiated AECs need first to be fully defined in health. In particular, the transition of the undifferentiated monolayer to the fully differentiated epithelium must be well characterised over time. While the differentiation of human^27,39^ and ovine^55^ AECs over time has been previously studied, to date there has been no such characterisation of the temporal differentiation of bovine AECs. We have previously established optimal culture conditions for the growth and differentiation of bovine bronchial epithelial cells (BBECs) in a serum-free defined defined medium^56^. In the present study, we aimed to characterise the differentiation of BBECs grown at an ALI over a period of 42 days from introduction of the ALI. Differentiation was assessed at three-day intervals from three days prior to the establishment of an ALI until day 42 post-ALI. Key markers of epithelial cell differentiation, including morphology, the formation of tight-junctions, and the presence of basal, ciliated and goblet cells, were assessed at each time-point. Furthermore, evidence of de-differentiation and deterioration of the cultures was also sought. In this way, an optimum window of AEC differentiation was identified which will allow for infection and other studies to be performed.

## Results

### General epithelial composition and morphology

**Figure 1:**
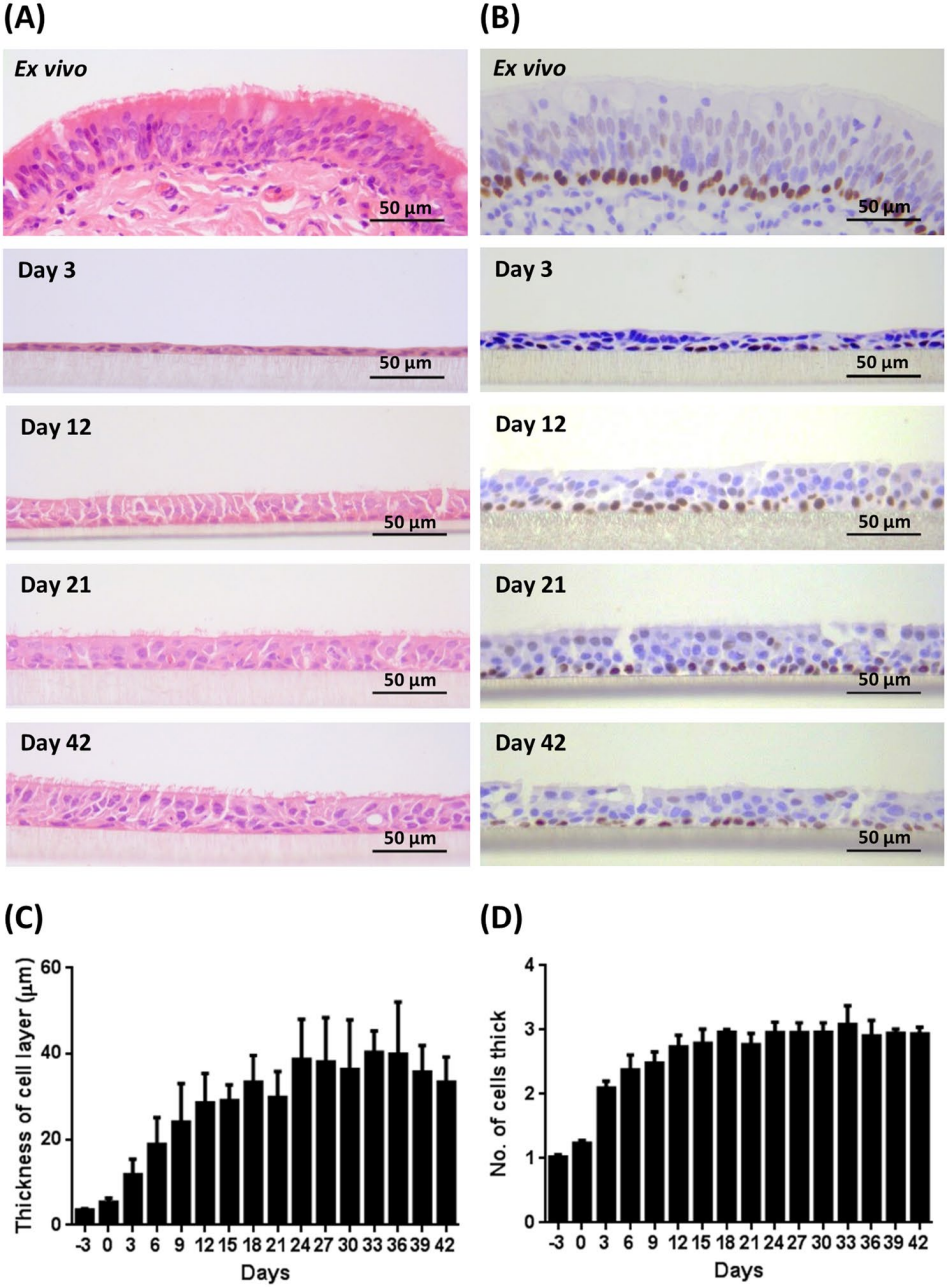
Histological assessment of BBEC differentiation over time. Bronchial epithelial cell cultures were grown for the indicated number of days at an ALI (relative to the establishment of the ALI), fixed and paraffin embedded using standard histological techniques. Samples of *ex vivo* bronchial epithelial tissue were similarly fixed and embedded. Sections were cut, deparaffinised and stained using (**A**) H&E and (**B**) immunohistochemistry with an anti-p63 antibody labelling the nuclei of basal cells (brown staining). Representative images are shown of *ex vivo* bronchial epithelium and BBECs grown for 3, 12, 21 and 42 days post-ALI (see also Figs S1 and S2). Quantitative analysis (using ImageJ) was performed of histological sections of BBEC layers fixed at three-day intervals ranging from day −3 to day 42 post-ALI (see Fig. S1); the epithelial thickness (**C**) and number of cell layers (**D**) comprising the epithelium were measured. For each insert, three measurements were taken (left, centre and right) in each of five randomised 400x fields of view evenly distributed across the strand; three inserts were analysed per time-point and the data represents the mean +/−standard deviation from tissue derived from three different animals (n = 9). Ordinary One-way ANOVA statistical analyses with post-test for linear trend demonstrated significant (P ≤ 0.05) increasing trends over time for both epithelial thickness (**C**) and the number of cell layers (**D**).

Bovine bronchial epithelial cells were cultured at an ALI for 42 days. Morphological assessment of the changes in epithelial structure over time was conducted on histological sections of samples fixed at three-day intervals, ranging from day −3 until day 42 post-ALI. Changes in the general morphology of the epithelial cell layer over time were assessed using haematoxylin and eosin (H&E) staining (Figs 1A and S1) and compared with that of *ex vivo* tissue (Fig. 1A). During the submerged growth phase (day −3 to day 0), the BBECs formed a squamous monolayer and exhibited no evidence of polarisation (Fig. S1). However, the establishment of an ALI and introduction of growth factors at day 0 initiated differentiation of the cultures which transitioned to pseudostratified epithelia that were reminiscent of *ex vivo* tissue (Figs 1A and S1). At day 3 post-ALI, the BBECs still exhibited squamous morphology but by day 12 post-ALI they had differentiated into a double layer with cells displaying cuboidal morphology (Fig. 1A). By day 21, the cultures were almost three cells in depth and the epithelia had become increasingly columnar and pseudostratified; thereafter, the epithelial morphology remained consistent until day 42 (Fig. 1A). From day 21 onwards, the morphology of the epithelial layer closely resembled that of *ex vivo* tissue in that it was columnar and pseudostratified albeit somewhat thinner (Fig. 1A). The epithelial cell layer gradually increased in thickness to a maximum of 30–40 µm between days 0 and 24 post-ALI and remained relatively constant thereafter (Fig. 1C). Similarly, the number of cells within the epithelial layer increased from day 0 post-ALI, reached a maximum at day 18 of 3.0 cells thick, and remained relatively constant thereafter (Fig. 1D). For comparison, the *ex vivo* epithelium (average for three animals) was 64.8 µm and 3.4 cells thick (results not shown). The transcription factor p63 is highly expressed within the nuclei of basal cells associated with epithelial tissues and is commonly used as a specific marker of this progenitor cell type^14,39,57^. Basal cells (with brown-staining nuclei) constituted a well-defined, single continuous layer attached to the basement membrane within the *ex vivo* tissue (Fig. 1B). Basal cells were present at day 3 in the BBEC cultures and they similarly formed a distinct single layer at the interface between the epithelium and insert membrane from day 9 until day 42 post-ALI (Figs 1B and S2).

The BBEC cultures were assessed for cellular and tissue deterioration following extended culturing at an ALI. There was a time-dependent increase in both the numbers of pyknotic nuclei (Fig. S3A,C) and vacuoles/gaps (Fig. S3B,D) within the epithelial layer. However, this trend correlated with an overall increase in the numbers of cells present in the epithelium over time. Once the number of cells within the epithelium had reached a peak at day 18 post-ALI (Fig. 1D), there was no subsequent significant increase in either the number of pyknotic cells or vacuoles/gaps (Fig. S3C,D; Ordinary one-way ANOVA). These findings indicated that the BBECs were stable for at least six weeks when cultured at an ALI.

**Figure 2:**
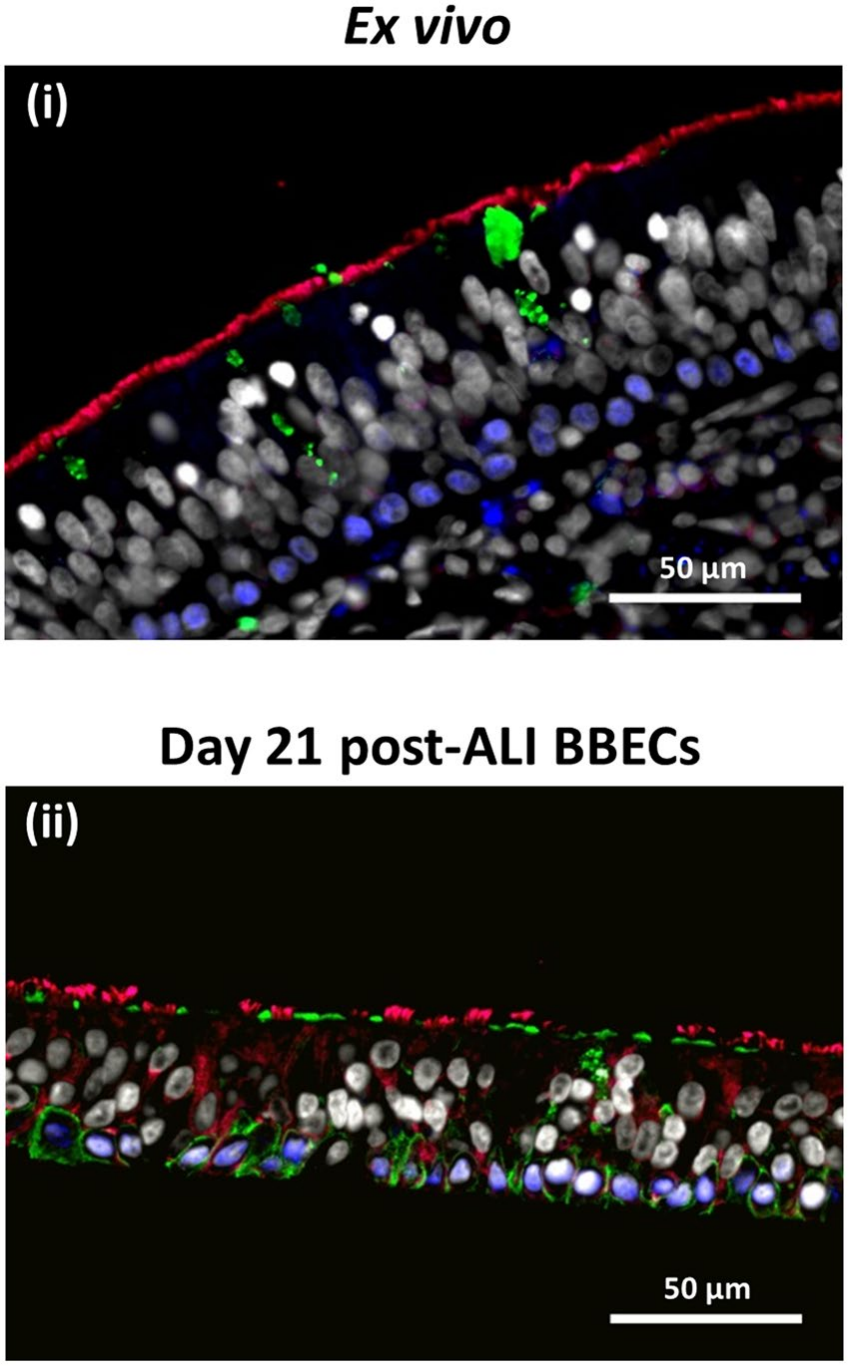
Comparison of cellular composition, morphology and polarisation of differentiated BBECs and *ex vivo* bronchial epithelium using fluorescent immunohistochemistry. *Ex vivo* bronchial epithelium dissected from the donor animal before cell extraction (i) and day 21 post-ALI BBEC cultures (ii) were fixed and paraffin-embedded using standard histological techniques. Sections were cut, deparaffinised and immunohistochemically stained to identify markers of specific epithelial cell-type as follows: cilia (ciliated cells) - red; Muc5AC (mucus and goblet cells) - green; p63 (basal cells) - blue; nuclei - grey.

Immunohistochemical staining of tissue sections was also performed to assess and compare the presence and distribution of key cell types in the BBEC layer with respect to that of *ex vivo* tissue (Fig. 2). All of the major epithelial cell types, including ciliated, goblet and basal cells, were identified within the BBEC layer (Fig. 2(ii)) and their distribution closely resembled that of the cells within the *ex vivo* tissue (Fig. 2(i)). β-tubulin-labelled ciliated cells (red) and Jacalin-labelled goblet cells or mucus (green) were present at the apical aspects of both epithelia whereas p63-labelled basal cells (blue) were present as a single layer at the base of the epithelium in both the BBEC cultures and *ex vivo* tissue.

### Barrier function and junctional integrity

**Figure 3:**
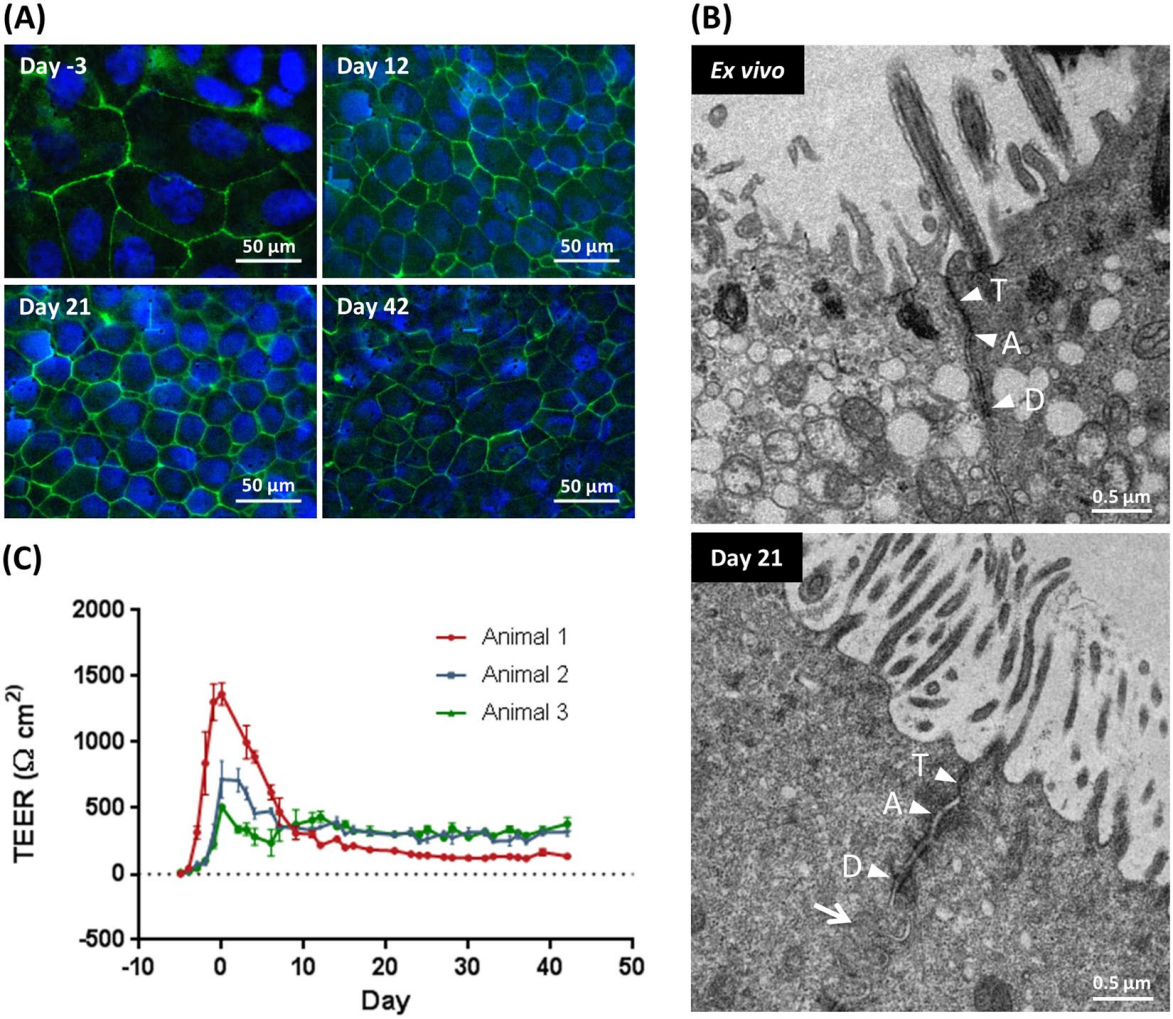
Tight-junction formation and changes in barrier function in BBEC cultures over time. Bronchial epithelial cell cultures were grown for the indicated number of days at an ALI (relative to the establishment of the ALI) and fixed *in situ* on the membranes. In (**A**) tight-junction formation was subsequently assessed using immunofluorescence staining of the tight-junction protein ZO-1 (tight junctions - green; nuclei - blue). Representative images are shown of BBECs at −3, 12, 21 and 42 days post-ALI (see also Fig. S4). In (**B**) junctional complexes of *ex vivo* bronchial epithelial tissue and day 21 post-ALI BBEC cultures were assessed by TEM. Tight-junctions (T), adherens junctions (A) and desmosomes (D) were present along the apicolateral borders of epithelial cells in both the *ex vivo* tissue and BBEC cultures. In addition, membranous interdigitations (arrow) were observed between adjacent cells in the BBEC cultures. In (**C**) tight-junction integrity during the course of epithelial cell proliferation and differentiation was assessed by measuring the TEER of BBEC cultures until day 42 post-ALI. Nine inserts were analysed per time-point and the data represents the mean +/−standard deviation from tissue derived from three different animals (n = 27).

The barrier function and junctional integrity of the BBECs was similarly assessed during culture over 42 days at ALI (Figs 3 and S4). Using the junctional protein ZO-1 as a marker, tight-junctions were present within the BBEC cultures at all time-points, during both submerged and ALI growth (Figs 3A and S4). During submerged growth (days −3 to 0) the cells were large and squamous but, once the ALI was established, the BBECs adopted a more cobblestone appearance reminiscent of differentiated epithelia and the number of cells with well-formed tight-junctions present per field of view increased (Figs 3A and S4). The intensity of ZO-1 staining remained consistent over the 42 days of ALI growth indicating that tight junction integrity remained intact and constant over the duration of the time-course. Furthermore, confocal imaging demonstrated that ZO-1 was localised to the sub-apical regions of the cell-to-cell borders, indicating correct location of tight-junctions within the epithelial cell cultures (Movie S1). In addition to tight junctions, transmission electron microscopy (TEM) identified adherens junctions and desmosomes at the expected locations within both day 21 post-ALI cultures and *ex vivo* tissue (Fig. 3B, arrowheads), further confirming the presence of junctional complexes and barrier integrity within the BBEC cultures. Further interactions between adjacent epithelial cells in the 21-day cultures included the presence of membranous interdigitations (Fig. 3B, arrow).

The transepithelial electrical resistance (TEER) of the BBEC cultures was measured daily during the submerged phase of growth and every 2 to 3 days after introduction of the ALI to confirm that the barrier function of the epithelium was formed and maintained (Fig. 3C). The BBECs reached confluency after two to three days of submerged growth as indicated by the establishment of a TEER in excess of ~200 Ω.cm^2^. Once confluency was achieved, the TEER increased rapidly and peaked after approximately five days of submerged culture (day 0 post-ALI); this coincided with the formation of intact barrier function since no leakage of media occurred from the basal compartment when the ALI was introduced. However, variation occurred in the peek TEER values (ranging from ~500 to1400 Ω.cm^2^) of cultures derived from different donor animals (Fig. 3C). Once an ALI was established, the TEER gradually declined during the ALI phase of growth until ~day 9 post-ALI at which point the TEER stabilised at ~150–300 Ω.cm^2^. Notably, the reduction in TEER between days 0 and 9 post-ALI did not coincide with a decrease in intensity of ZO-1 staining over the same time-period (Fig. S4) and the barrier function remained intact for the duration of the 42-day period of ALI culture.

### Ciliogenesis

**Figure 4:**
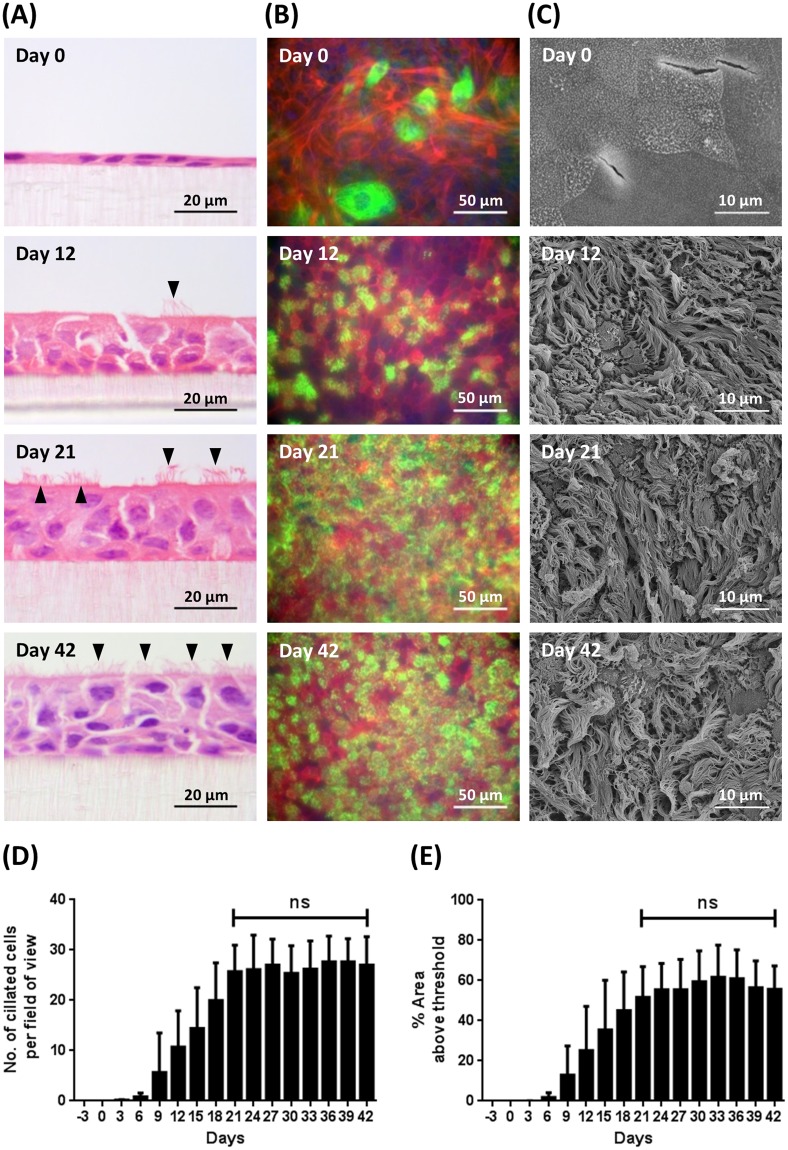
Cilia formation in BBEC cultures over time. Bronchial epithelial cell cultures were grown for the indicated number of days at an ALI (relative to the establishment of the ALI) before fixation. The BBEC cultures were subsequently processed to assess ciliation using (**A**) H&E staining of histological sections (arrowheads indicate ciliated cells), (**B**) immunofluorescence staining (cilia - green; F-actin - red; nuclei - blue) and (**C**) SEM. Representative images are shown of BBECs grown for 0, 12, 21 and 41 days post-ALI (see also Figs S1, S5 and S6). Quantitative analysis (using ImageJ) of ciliation of BBEC cultures fixed at three-day intervals ranging from day −3 to day 42 post-ALI was performed (**D**) by counting the number of ciliated cells per field of view in H&E-stained sections (see Fig. S1) and (**E**) using fluorescence intensity thresholding of immunostained cultures (see Fig. S5). In (**D**) for each insert, ciliated cells were counted in each of five randomised 400x fields of view evenly distributed across the strand. In (**E**) ciliation was quantified by measuring the area above a fluorescence intensity threshold in ImageJ; for each insert, five regions evenly distributed across the sample were measured. For all of the above quantifications, three inserts were analysed per time-point and the data represents the mean +/−standard deviation from tissue derived from three different animals (n = 9). Statistical significance was tested using an Ordinary one-way ANOVA: ns = not significant.

The temporal development of cilia on the apical surface of the BBEC cultures was assessed in H&E-stained histological sections (Figs 4A and S1), by immunofluorescence microscopy (Figs 4B and S5) and by scanning electron microscopy (SEM) (Figs 4C and S6). Histological analysis revealed an absence of cilia at day 0 post-ALI (Fig. 4A), the appearance of small numbers of short cilia at days 6 and 9 (Fig. S1), the presence of fully-formed cilia by day 12 (Fig. 4A, arrowhead) and an increasing number of ciliated cells thereafter (Fig. 4A, arrowheads). Quantitative assessment of the numbers of ciliated cells present in H&E-stained sections demonstrated a steady increase from day 6, with numbers peaking between days 21 and 27 and remaining relatively constant thereafter (Figs 4D and S1). Immunofluorescence microscopy of the apical surface revealed the presence of intracellular β-tubulin (presumably associated with cytoskeletal microtubules), but no cilia, at day 0 post-ALI (Fig. 4B). Cilia were relatively abundant by day 12 with large numbers of individual “tufts” of cilia apparent; cilia covered most of the apical surface by day 21 and there was little change by day 42 (Fig. 4B). Quantitative assessment of ciliation by immunofluorescence microscopy demonstrated a steady increase in the numbers of cilia from day 6 until day 24, after which the numbers of cilia remained relatively constant (Figs 4E and S5). Using both approaches, there was no significant increase in the number of ciliated cells between days 21 and 42 post-ALI (Fig. 4D,E; Ordinary one-way ANOVA). Scanning electron microscopy confirmed the absence of cilia at day 0 and the presence of abundant cilia at days 12, 21 and 42 (Fig. 4C). Indeed, the process of ciliogenesis was clearly apparent using SEM with patches of short cilia present at day 6 which progressively increased in length at days 9 and 12 (Fig. S6). The overall appearance of ciliated cells subsequently changed very little from day 12 onwards although there was a gradual increase in the percentage surface area covered following the trend shown in Fig. 4D,E. However, ciliation of the differentiated BBEC cultures did not achieve the uniformity and density of that observed in *ex vivo* tissue (Fig. S6).

### Mucus production

**Figure 5:**
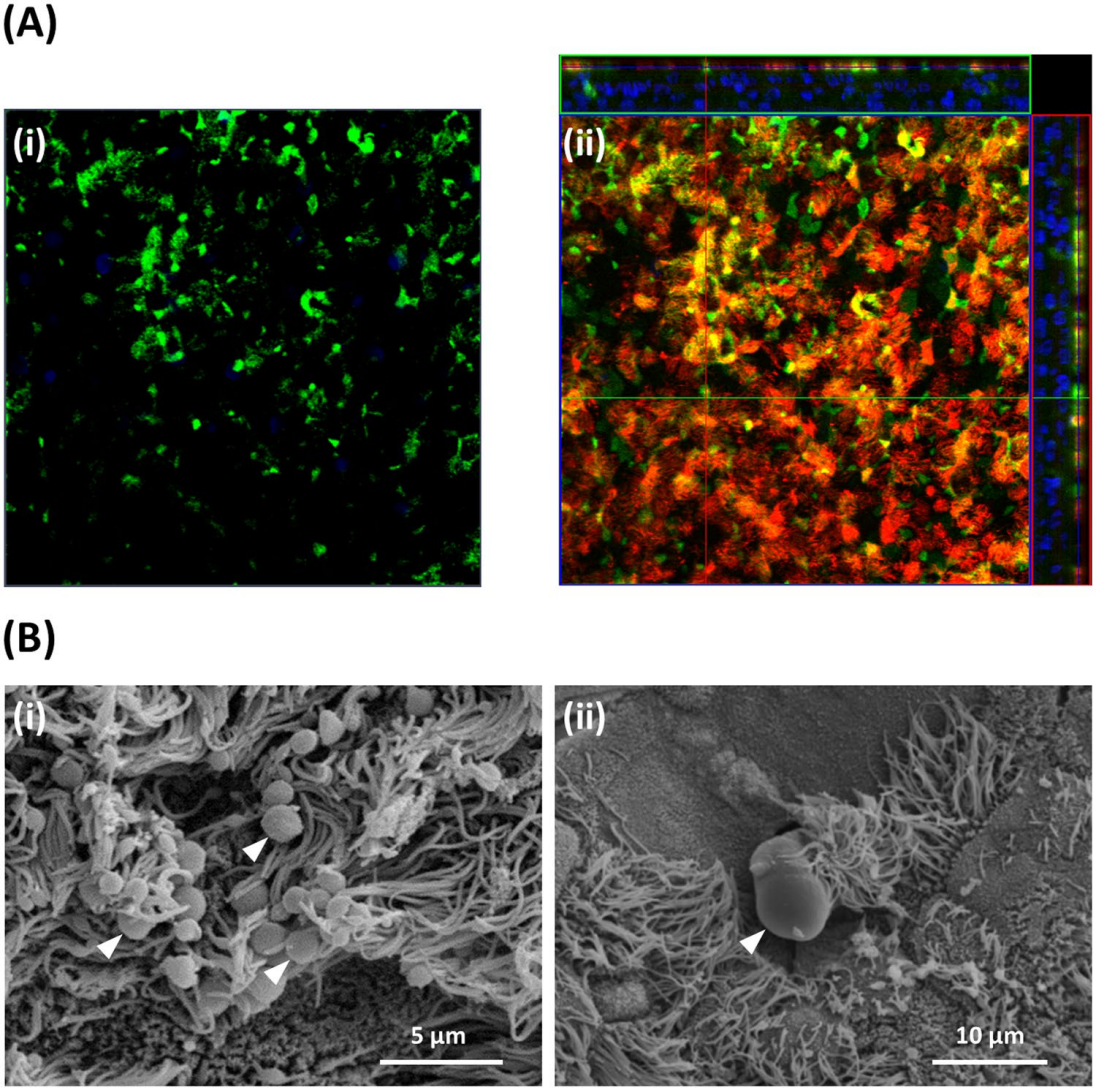
Mucus production and identification of goblet cells in BBEC cultures. Bronchial epithelial cell cultures were grown for 21 days at an ALI before fixation. The BBEC cultures were subsequently processed to assess mucus production and identify goblet cells using (**A**) immunofluorescence microscopy and (**B**) SEM. In (**A**) standard immunofluorescence imaging shows mucus and its association with goblet cells (i) (mucus [Muc5AC] - green; nuclei – blue); Z-stack orthogonal representation highlights surface-localisation of mucus (ii) (mucus [Muc5AC] - green; cilia - red; nuclei - blue). In (**B**) SEM revealed the presence of numerous globules of mucus and their close association with cilia ([i], arrowheads) and identified goblet cells in the act of extruding mucus ([ii], arrowhead).

The development of goblet cells and mucus production during the differentiation of the BBECs over the 42-day period of ALI growth was assessed by periodic acid-Schiff (PAS)-staining of histological sections (Fig. S7). Goblet cells were readily identified in *ex vivo* tissue but were observed less frequently within differentiated BBECs (Fig. S7, arrowheads). There was evidence of goblet cells or mucus production from early time points within the BBECs but a progressive increase in goblet cell maturation over time was not observed. Well-defined goblet cells and/or secreted mucus were observed at days 18 and 21 whereas at later time-points (days 33 to 42) mucus appeared to be associated more frequently with epithelial vacuoles/gaps (Fig. S7, arrowheads) possibly resulting from structural disruption of occasional goblet cells. Jacalin-labelling of BBEC cultures (Fig. 5A) together with SEM (Fig. 5B) provided more conclusive evidence of goblet cells and mucus production. Jacalin is a lectin which specifically binds to the Muc5AC component of mucus and goblet cells (Fig. 5A(i)) and functions as a marker of goblet cells^40,58^. There was evidence for the association of Muc5AC with goblet cells at all time points although the cells were smaller and present in higher numbers at later time-points compared with earlier time-points (Fig. S8). The surface-localisation of Jacalin-staining highlighting the presence of goblet cells in differentiated BBECs at 21 days is clearly observed by confocal imaging (Fig. 5A(ii)). Secreted mucus was observed by SEM in cultures from day 15 post-ALI often in the form of globules that were frequently associated with cilia (Fig. 5B(i), arrowheads) or sometimes as a web-like coating of the apical surface (results not shown). Occasionally, goblet cells were observed in the process of actively extruding mucus (Fig. 5B(ii), arrowhead).

### Ultrastructural detail

**Figure 6:**
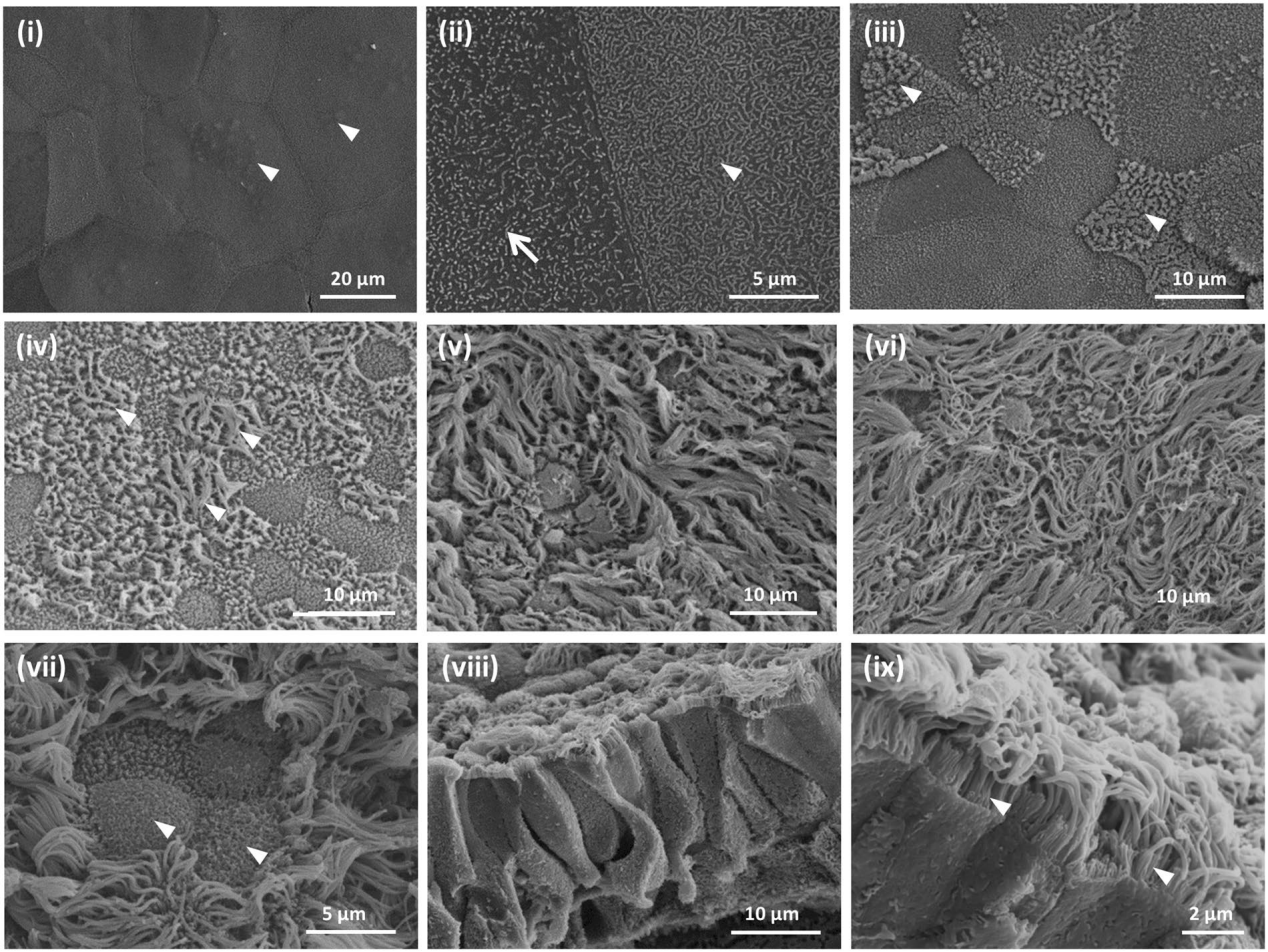
Scanning electron microscopic assessment of ultrastructural changes in BBEC cultures over time. Bronchial epithelial cell cultures were grown until 42 days post-ALI and samples fixed at three day intervals and processed for SEM (see also Fig. S6). Representative micrographs show (i) the undifferentiated, flattened apical surface at day 0 post-ALI with extruding nuclei (arrowheads); (ii) microvilli (arrow) and microplicae (arrowhead) on undifferentiated cells at day 0 post-ALI; (iii) longer microvilli (arrowheads) on day 3 post ALI cells undergoing differentiation; (iv) early stages of cilia formation (arrowheads) on day 6 post-ALI cells undergoing differentiation; (v) numerous well-formed cilia on day 12 post ALI cells; (vi) maximum ciliation on day 24 post-ALI cells; (vii) goblet cells (arrowheads) present in day 21 post-ALI cultures; (viii) section of day 18 post-ALI epithelium showing pseudostratified structure; and (ix) microvilli (arrowheads) at base of cilia associated with day 36 post-ALI cells.

Ultrastructural changes during the course of differentiation of the BBECs were assessed at three-day intervals by SEM (Figs 6 and S6). During the submerged phase of growth, the cells possessed a squamous morphology often with protruding nuclei visible (Fig. 6(i), arrowheads). At this stage, the cells were devoid of markers of differentiation such as cilia and other features although the cell boundaries were clearly demarcated. At higher magnification, distinct surface features such as microvilli (arrow) and microplicae (arrowhead) were apparent (Fig. 6(ii)). Within three days of introducing the ALI, the cells had a more defined cobblestone morphology and the microvilli were denser and more pronounced; indeed, these extended microvilli marked the early stages of ciliogenesis (Fig. 6(iii), arrowheads). The key change over the following days was the onset of ciliogenesis; clumps of short cilia were visible by day 6 post-ALI (Fig. 6(iv), arrowheads) and ciliation was well-developed by day 12-post-ALI (Fig. 6(v)) with maximum development and coverage by day 24 (Fig. 6(vi)). Goblet cells, either singly or as clusters, were frequently observed amongst the cilia and these often possessed a highly microvillous surface (Fig. 6(vii), arrowheads). Exposed cross-sections of the cell layer at day 18 post-ALI revealed a stereotypical pseudostratified morphology (Fig. 6(viii)). Most of the cells at the apical aspects of these fully differentiated cultures were ciliated although microvilli were observed at the bases of the cilia (Fig. 6(ix), arrowheads). There were no significant changes in the overall topography of the epithelial cell surface between days 21 and 42 post-ALI as discerned by SEM; in particular, there were no signs of degradation or de-differentiation of epithelial cells over the course of the 6 weeks of culture (Fig. S6).

**Figure 7:**
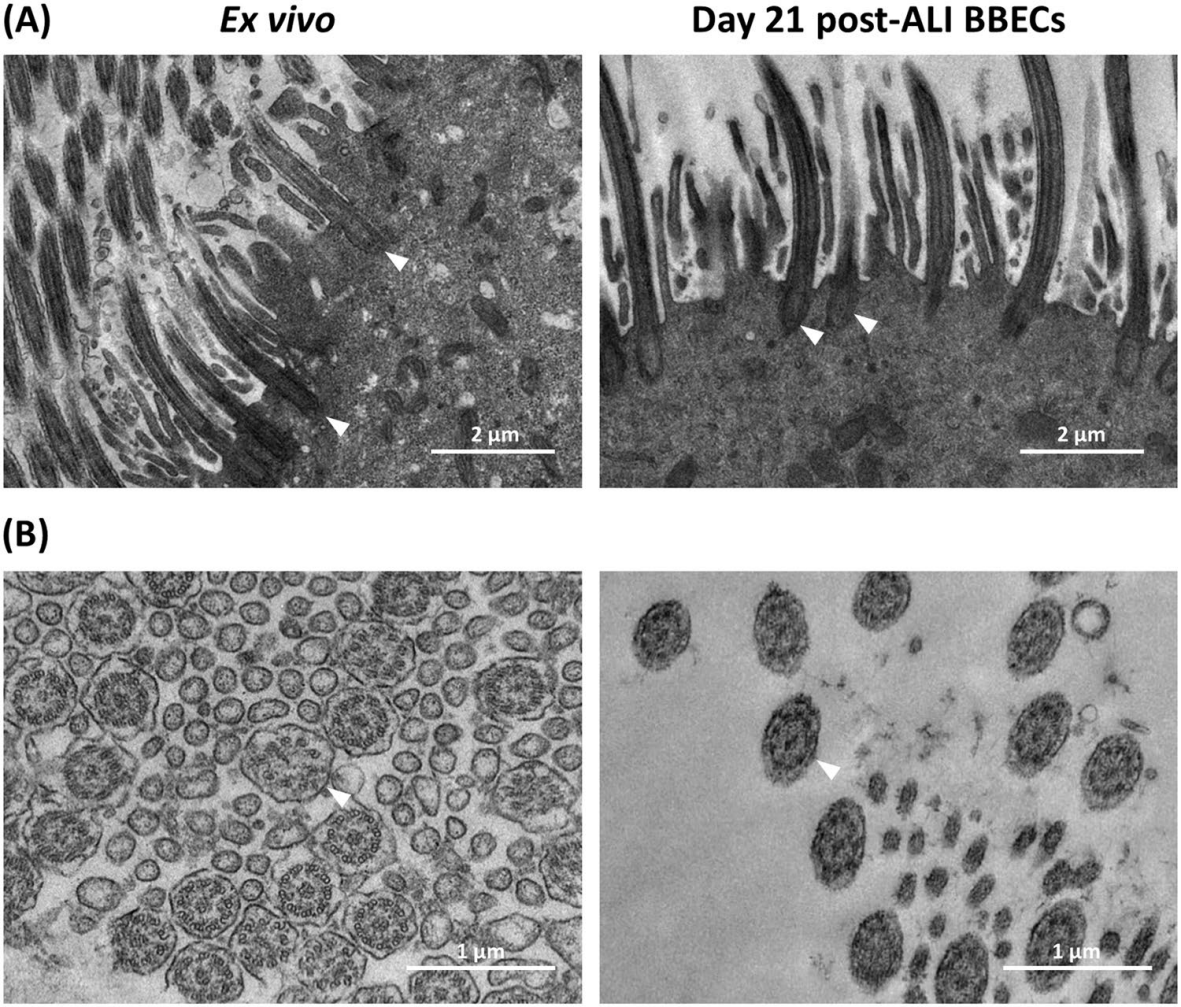
Ultra-structural comparison of cilia formation in *ex vivo* bronchial epithelium and differentiated BBECs using TEM. *Ex vivo* bronchial epithelium and day 21 post-ALI BBEC cultures were fixed and resin embedded, and ultrathin sections were cut and contrast stained. Comparisons are made of (**A**) longitudinal sections of cilia and basal bodies (arrowheads) and (**B**) transverse sections of cilia showing 9 + 2 axoneme arrangement (arrowheads).

**Figure 8:**
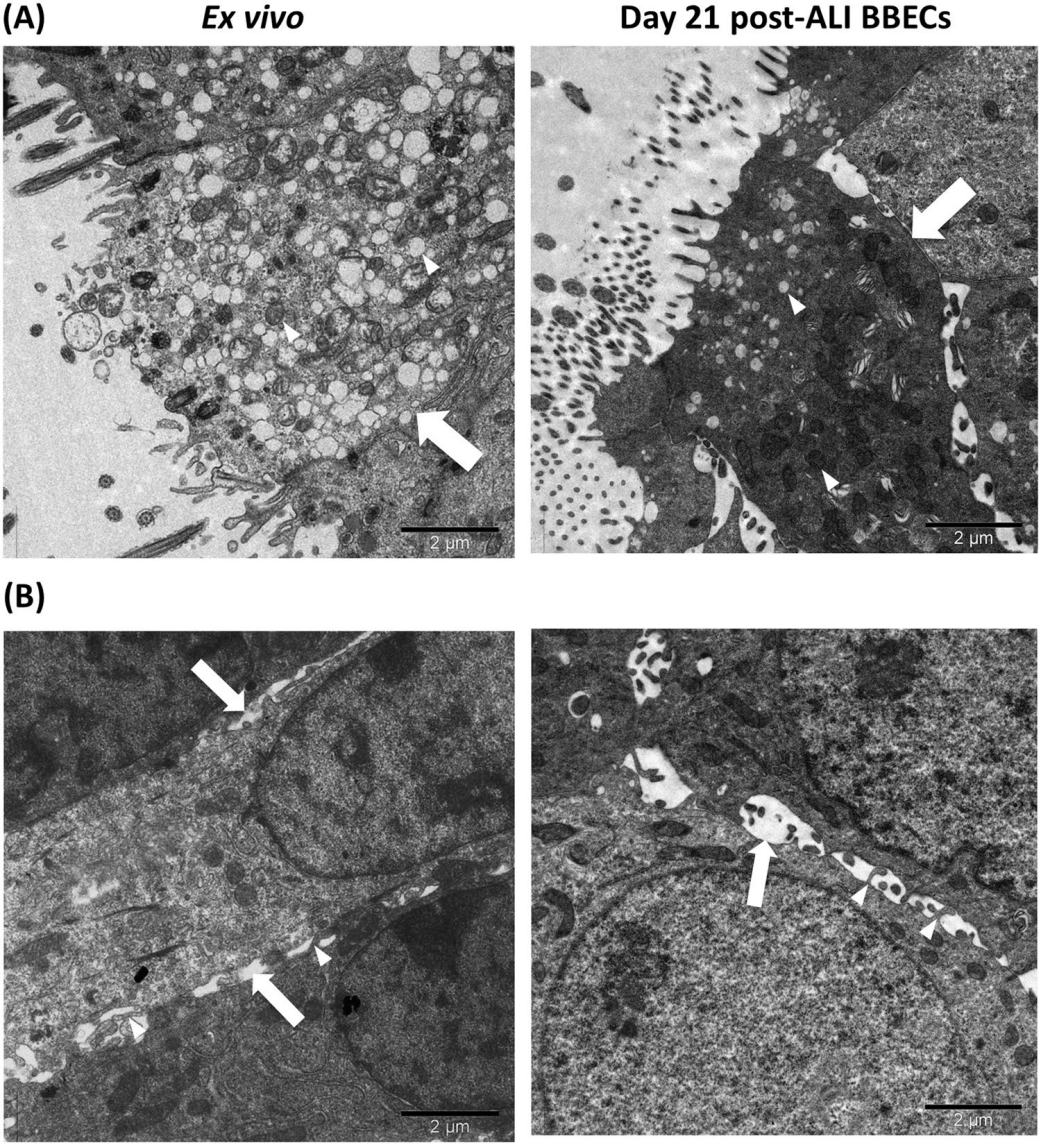
Ultra-structural comparison of goblet cells and paracellular spaces in *ex vivo* bronchial epithelium and differentiated BBECs using TEM. *Ex vivo* bronchial epithelium and day 21 post-ALI BBEC cultures were fixed and resin embedded, and ultrathin sections were cut and contrast stained. Images highlight (**A**) goblet cells (arrows) containing numerous secretory vesicles (arrowheads) and (**B**) paracellular spaces (arrows) containing membranous interdigitations (arrowheads).

Further ultra-structural comparisons of day-21 post-ALI BBEC cultures and *ex vivo* tissue were made by TEM (Figs 7 and 8). The ultrastructure of the cilia associated with BBEC cultures and *ex vivo* tissue was very similar (Fig. 7). Thus, the configurations of both the cilial basal bodies (Fig. 7A, arrowheads) and the 9 + 2 axoneme arrangements (Fig. 7B, arrowheads) were highly consistent in each case; there was no indication of cilia malformation in differentiated BBECs. In support of these observations, bright-field microscopy of differentiated BBECs to which microspheres had been added demonstrated cilia beating in a coordinated fashion, thus confirming their functionality (Movie S2). Large numbers of goblet cells (arrows) containing numerous secretory vesicles (arrowheads) were present within the *ex vivo* tissue; cells of similar appearance were also identified within the BBEC layer although these were less numerous and contained fewer vesicles (Fig. 8A). Paracellular spaces were observed between adjacent cells within both the *ex vivo* tissue and BBEC layer (Fig. 8B, arrows) and, within these spaces, membrane interdigitations were visible that appeared to be associated with the adjacent cells (Fig. 8B, arrowheads).

## Discussion

Having previously established optimum culture conditions for the differentiation of BBECs at an ALI^56^, the present study aimed to characterise the proliferation and differentiation of these cells at three-day intervals over a 42-day period (post-ALI) with a view to identifying a window during which the cultures were optimally differentiated. The major cell types normally associated with *in vivo* airway epithelium (i.e. ciliated, goblet and basal cells) were present within the differentiated BBEC cultures and these were replicated in a pseudostratified morphology comparable to that of *ex vivo* tissue (Figs 1 and 2). Importantly, the epithelium exhibited no evidence of major degradation or de-differentiation during the 42 days of ALI culture; there was no reduction in the number of ciliated cells (Fig. 4), no significant increase in the number of pyknotic cells or epithelial vacuoles/gaps (Fig. S3), and no adverse changes visible by SEM (Figs 6 and S6) by day 42 post-ALI. However, at later time-points (days 33 to 42 post-ALI) the detection of PAS-positive mucus in association with epithelial vacuoles/gaps could suggest a mild exent of time-dependent disruption of goblet cells in the culture system. These observations were in marked contrast to the findings of Prytherch *et al*. (2011) who described rapid deterioration and de-differentiation of human bronchial epithelial cells (HBECs) after 33 days of culture.

The formation of junctional-complexes (which include tight junctions, adherens junctions and desmosomes) between cells creates a physicochemical barrier against inhaled particulates and prevent the penetration of microorganisms and chemicals into the interstitial compartment^3,5,59^. Tight junctions are involved in the regulation of solute and ion transport across epithelia and are localised at the apicolateral border of individual airway epithelial cells^3,5,60^. Certain bacterial^41,61,62^ and viral^42,63^ pathogens specifically target and degrade the tight junctions of airway epithelia during paracellular infection processes. Therefore, the presence of normal, functioning tight junctions is an essential feature of differentiated AECs to allow for representative modelling of respiratory tract infections. These structures were observed to be present at the apicolateral borders of epithelial cells in both the differentiated BBEC cultures and *ex vivo* tissue by TEM (Fig. 3B); their apicolateral location in the BBECs was also confirmed by confocal microscopy (Movie S1). Adherens junctions and desmosomes are involved in cell-to-cell adhesion and attachment and play important roles in maintaining the integrity of respiratory epithelium^3,64–66^. These structures were also observed between epithelial cells in both the BBEC cultures and *ex vivo* tissue by TEM (Fig. 3B).

Transepithelial resistance is an important parameter for assessing the integrity of the junctional complexes between cells^67^ and the observed trend in TEER for the BBECs over time (Fig. 3C) was similar to the pattern observed in AEC cultures derived from other animal species^37,55,68^. The variation in TEER over the 42-day course of ALI culture was not reflected by changes in the distribution or amount of the tight-junctional protein ZO-1, which was maintained at a stable level throughout the duration of the time-course (Figs 3A and S4). The ZO-1 protein was localised at the cell-cell borders at all time-points suggesting that the tight-junctions were formed within the BBEC cultures during the submerged growth phase and remained stable during differentiation at an ALI; similar observations were described in ovine^55^ and equine^68^ AECs grown at an ALI. In contrast, ZO-1 took up to 15 days to migrate to the cell periphery and appeared to decline between days 33 and 42 post-ALI in HBECs cultured at an ALI^39^. Taken together, our data indicate that differentiated BBECs possess stable junctional complexes (tight and adherence junctions, and desmosomes) and maintain intact barrier function for up to 42 days of ALI culture. These findings will be important in terms of using differentiated BBECs to investigate potential routes and mechanisms of infection by bovine respiratory tract pathogens^41,42,61–63^.

A highly ciliated apical surface is one of the hallmarks of a healthy, functioning airway epithelium; ciliated cells function in conjunction with mucus production to ensure removal of invading pathogens and inhaled particles through the process known as mucociliary clearance^6–8,69^. The presence of actively functioning cilia is characteristic of differentiated AEC models representing various animal species^36,55,68,70^. More importantly, the presence of cilia is an absolute requirement for the use of such models in infection studies because many bacterial^23,24,27,71,72^ and viral^20–22,28,73^ pathogens specifically target cilia or ciliated cells during infection. Quantitative assessment of ciliogenesis during BBEC differentiation (Fig. 4) demonstrated that cilia were present from as early as day 6 post-ALI, which was sooner than has been reported in previous models^27,39^. Ciliated cells increased in abundance until ~day 21 post-ALI, at which point most of the apical aspect of the cultures was composed of ciliated cells. The observed increase in the number of ciliated cells during prolonged culture at an ALI was expected because it has also been observed during differentiation of AECs from other species^27,39,55,74,75^. However, the density and uniformity of ciliation of fully differentiated BBEC cultures was generally less than that of *ex vivo* tissue (Fig. S6) and this finding is in agreement with that described for differentiated HBECs and native tissue^40^. Ultrastructural analysis of ciliated cells by SEM and TEM demonstrated that cilia associated with differentiated BBECs were of comparable morphology to those of *ex vivo* tissue, both in terms of the structure of the basal bodies and the 9 + 2 arrangement of axonemes (Fig. 7)^36,39,69,76^. We also observed, with the aid of microspheres, vigorous beating of cilia in an apparently co-ordinated manner (Movie S2) and as described in other ALI systems^36,70,77,78^, further demonstrating that differentiated BBECs have the potential for active mucociliary clearance.

Together with cilia, mucus is the second major component associated with the mucociliary function of airway epithelia and, in native epithelium, is secreted by goblet cells and from submucosal glands^2,3,79^. The secretion of mucus onto the apical surface of differentiated AECs is also an important requirement for the use of such models in infection and similar studies. Goblet cells and mucus production have previously been described in differentiated AECs originating from humans^40^ and various animal species^31,75,80^. Furthermore, proteomic analysis has demonstrated that mucus secretions from differentiated AECs grown at an ALI are highly representative of natural *in vivo* secretions^81^. In the present study, evidence from PAS-stained histological sections (Fig. S7) and Jacalin-labelled cultures (Fig. S8) demonstrated the presence of goblet cells and the production of mucus from early stages of BBEC differentiation. The identification by SEM of mucus globules on the apical surface of BBEC cultures and goblet cells actively extruding mucus (Fig. 5B) confirmed these findings. Although goblet cells and mucin were present from the very early stages of BBEC differentiation, extensive secretion of mucus onto the epithelial cell surface seemed to be associated with well-differentiated (i.e. older than 15 days) cultures.

Basal cells represent an important component of airway epithelia because they function as progenitor (stem) cells and are involved in repair and regeneration following injury^14,82,83^. However, basal cells are also the target cell of certain pathogens^28,84,85^ and may be directly involved in the infection process. For these reasons, basal cells also represent important components of differentiated AEC models to be used in infection studies. Basal cells were identified using the marker p63^39,55,85,86^ and were observed within the epithelium at all time-points examined, forming a continuous layer along the basal aspect (Figs 1B and S2). This distribution mimicked that observed by ourselves in *ex vivo* tissue (Fig. 1B), in which a layer of basal cells was attached to the basement membrane, as well as by others in both native airway epithelia and differentiated AECs of various animal species^55,86,87^. The number of basal cells within the epithelium remained constant over the duration of the time-course and regardless of the differentiation state of the epithelium. The similar distribution of basal cells within the differentiated BBEC layer to that of *ex vivo* tissue is important because it suggests that the epithelium has the potential for tissue repair and regeneration^83,85^. Indeed, we have demonstrated epithelial regeneration following injury using a scratch assay (data not shown) and this property will be important in tissue repair during recovery from infection and other injury.

A major aim of the present study was to identify a window during which the BBEC cultures were optimally differentiated to allow infection experiments to be performed. The identification of such a window has important implications for the use of differentiated BBECs for infectious disease research because the degree of differentiation of AECs can influence the ability of both bacterial^27,88^ and viral^20,89^ pathogens to colonise the respiratory epithelium. The differentiation state of AECs can also affect the pro-inflammatory response of epithelial cells to both infectious and toxicological agents^89,90^. Importantly, our observations demonstrated that BBECs grown at an ALI became fully differentiated by day 21 and remained healthy until day 42 post-ALI. Thus, differentiated BBECs provide a stable window of at least 21 days during which long-term single pathogen or co-infection experiments, or repeat-exposure toxicology studies, can be performed. Further analyses of cultures derived from a single animal demonstrated that BBECs could be maintained for at least 112 days. However, such cultures appeared to undergo de-differentiation after ~day 45 because they were characterised by an increase in the number of cells comprising the epithelium, by an accompanying loss of pseudostratified structure and, most noticeably, by an almost complete loss of ciliation between days ~45 and 112 (Fig. S9).

In conclusion, we have characterised the proliferation and differentiation of primary BBECs grown at an ALI at three-day intervals over a period of 42 days from the introduction of the ALI. The differentiated BBEC model was highly representative of the *ex vivo* epithelium from which the epithelial cells were derived; it comprised the major epithelial cell types present in bronchial epithelium (i.e. ciliated, goblet and basal cells) and these formed a columnar, pseudostratified epithelium that was highly reflective of typical *in vivo* airway epithelium. In particular, the hallmark defences of the respiratory tract, namely barrier function and mucocilary clearance, were present thus ensuring that the model is an excellent mimic of the mucosal phenotype of the bovine respiratory tract. The model was fully differentiated by day 21 post-ALI and remained healthy and stable for a further 21 days; it also displayed limited inter-donor variation. Overall, our differentiated BBEC model provides an excellent mimic of the bovine respiratory tract; it has a three-week window during which infection or other experiments can be performed, and will have wide applications for infection, toxicology or general airway physiological research.

## Methods

### Isolation of bovine bronchial epithelial cells

Bronchial epithelial cells were isolated from the lungs of freshly-slaughtered cattle (obtained from Sandyford abattoir, Paisley, UK) aged 18–36 months as previously described^56^. Briefly, the lungs were transported to the laboratory on ice and, after swabbing for bacterial/fungal contamination, the left and right bronchi were dissected and sections incubated overnight at 4 °C in *digestion medium* (DM). Small (~1 cm^2^) pieces of bronchial tissue were also fixed in 2% (w/v) formaldehyde for histological analysis or in 1.5% (v/v) glutaraldehyde for SEM and TEM. Epithelial cells were recovered from the bronchial sections as previously described and resuspended in *submerged growth medium* (SGM) at a cell density of 5.0 × 10^5^ cells/ml. Ten-ml of cell suspension were seeded into T75 tissue culture flasks (5.0 × 10^6^ cells/flask) and incubated at 37 °C in a humidified atmosphere containing 5% CO_2_ and 14% O_2_.

### Culture of bovine bronchial epithelial cells at an air-liquid interface

The subsequent culture and differentiation of the BBECs was performed as previously described^56^. Briefly, the BBECs were grown until 80–90% confluent (~4 days), trypsinised, and seeded in SGM onto the apical surface of 12-mm diameter, PET Thincerts of 0.4 µm pore diameter and containing 1.0 × 10^8^ pores per cm^2^ (Greiner, #665640). The epithelial cells were cultured at 37 °C in a humidified atmosphere containing 5% CO_2_ and 14% O_2_ and the growth medium changed every 2 to 3 days as previously described. The TEER of the cultures was measured daily during the submerged phase of growth using an EVOM2 Epithelial Voltohmmeter (World Precision Instruments, UK) according to the manufacturer’s instructions. When the TEER reached 200 Ω.cm^2^ or above (at ~2 days), the growth medium was replaced with a 50:50 mixture of SGM and ALI medium. When the TEER reached 500 Ω.cm^2^ (at ~5 days, indicating successful barrier formation), an ALI was generated (this represented day 0 post-ALI) and the cells were fed exclusively from the basal compartment with ALI medium every 2 to 3 days until day 42 post-ALI as previously described. During the ALI phase of growth the TEER of the cultures was measured every 2 to 3 days at the time of media replenishment.

### Histology and immunohistochemistry

Bovine bronchial epithelial cell cultures were fixed at three-day intervals, from three days prior to the establishment of an ALI (day −3) until day 42 post-ALI, as previously described^56^. Fixed BBEC cultures, as well as *ex vivo* tissue samples, were subsequently processed, sectioned and stained with H&E or PAS using standard histological techniques. Immunohistochemical staining and counterstaining of basal cells was also performed using mouse anti-p63 antibody and Gill’s haematoxylin, respectively, as previously described^56^. Tissue sections were viewed with a Leica DM2000 light microscope.

### Quantification of features by light microscopy

Histological sections were stained with H&E and five randomised 400x fields of view evenly distributed across the strand were analysed as previously described^56^. The thickness of the epithelial cell layer and the number of cells forming the epithelium were determined at three points in each field of view using ImageJ as previously described^56^; the number of ciliated cells, epithelial vacuoles/gaps and pyknotic cells were also quantified.

### Fluorescent immunohistochemistry

Cultures were fixed, processed and sectioned as described above. Tissue sections were mounted onto microscope slides, deparaffinised by two 5 min washes in 100% xylene and rehydrated through a series of decreasing ethanol concentrations. The samples were subjected to heat-induced epitope retrieval by immersion in sodium citrate buffer (10 mM sodium citrate, 0.05% [v/v] Tween-20, pH 6) at 100 °C for 20 min. Non-specific binding sites were blocked by incubation in blocking buffer (PBS containing 0.05% [v/v] Tween-20, 10% [v/v] goat serum and 1% [w/v] bovine serum albumin) for 1 h at room temperature. The samples were incubated with primary antibody diluted 1:200 in blocking buffer for 1 h at room temperature, washed three times in PBS containing 0.05% (v/v) Tween-20 (PBST) for 2 min, and incubated with secondary antibody diluted 1:400 in blocking buffer for 1 h at room temperature. Cilia were detected with rabbit anti-β-tubulin antibody (Abcam; #ab6046) and visualised using goat anti-rabbit-Alexa Fluor 647 antibody (Thermo Fisher; #A-21244). Basal cells were detected with mouse anti-p63 antibody (Abcam, #ab735) and visualised with goat anti-mouse-Alexa Fluor 568 antibody (Thermo Fisher; #A-11031). Goblet cells were detected with fluorescein-labelled Jacalin (Vector Laboratories; FL-1151)^58^. After three 2-min washes in PBST, nuclei were stained with 300 nM 4′,6 diamidino-2-phenylindole (DAPI) for 10 min. The samples were washed three times with PBST, a drop of Vectashield mounting medium (Vector Laboratories) was added to the surface of each sample and a coverslip sealed in place using clear nail varnish. The tissue sections were observed with a Leica DMi8 immunofluorescence microscope and captured images analysed using ImageJ.

### Immunofluorescence microscopy

Cultures were prepared for immunofluorescence microscopy as previously described^56^. Briefly, the epithelial cells were permeabilised, non-specific binding sites blocked, and the samples incubated with anti-ZO-1 or -β-tubulin primary antibodies (followed by secondary antibodies) to stain tight-junctions and cilia, respectively; goblet cells were stained with fluorescein-labelled Jacalin, F-actin with rhodamine phalloidin and cell nuclei with DAPI. After staining, the membranes were cut from the inserts and mounted onto glass slides in Vectashield mounting medium (Vector Laboratories). Images were acquired with a Leica DMi8 immunofluorescence microscope and analysis of captured images performed using ImageJ software.

### Quantification of ciliogenesis

To quantify the degree of ciliation on the apical surface, five randomised fields of view of each β-tubulin-stained insert were acquired via a 20x objective. The percentage coverage of cilia was quantified using ImageJ as previously described^56^.

### Scanning electron microscopy

Cultures were fixed and processed for SEM as previously described^56^. Briefly, the samples were fixed in 1.5% (v/v) glutaraldehyde diluted in 0.1 M sodium cacodylate buffer, post-fixed in 1% (w/v) osmium tetroxide and stained with 0.5% (w/v) uranyl acetate. After dehydrating through a series of increasing ethanol concentrations to absolute ethanol, and subsequently in hexamethyldisilazane, the membranes were cut from the inserts, mounted onto aluminium SEM stubs and gold sputter-coated. The epithelium of fixed *ex vivo* bronchial tissue was cut from the underlying cartilage and processed as described above for the BBEC cultures. The BBEC cultures and *ex vivo* tissue were analysed with a Jeol 6400 scanning electron microscope at 10 kV.

### Transmission electron microscopy

Cultures were fixed and processed as described above for SEM until the dehydration stage in absolute ethanol. Subsequently, the samples were washed in propylene oxide three times for 10 min and immersed overnight in a 1:1 dilution of propylene oxide and Aridite/Epon 812 resin. After three changes of resin, the samples were embedded in resin within rubber moulds and allowed to polymerise at 60 °C for 48 h. Ultrathin (50 nm) sections of the resin-embedded samples were cut using a Leica Ultracut UCT ultramicrotome and a DiATOME diamond knife and mounted on 100 mesh Formvar-coated copper grids. The samples were contrast-stained with 2% (w/v) methanolic uranyl acetate for 5 min and Reynolds lead citrate for 5 min. The epithelium of fixed *ex vivo* bronchial tissue was cut from the underlying cartilage and processed as described above for the BBEC cultures. The BBEC cultures and *ex vivo* tissue were analysed on a FEI Tecnai transmission electron microscope at 200 kV and images captured with a Gatan Multiscan 794 camera.

### Data Analysis

All experiments were independently performed three times using epithelial cells derived from three individual donor animals. For quantitative analyses, three individual cultures from each donor animal were analysed (n = 9). Results are presented as the mean ± standard deviation. Data were statistically analysed using one-way ANOVAs for comparison of greater than three groups. Significance was determined by a *p*-value less than 0.05. Analyses were performed using GraphPad Prism (GraphPad Software Inc.).

## Electronic supplementary material

Supplementary information

Supplementary Movie S1

Supplementary Movie S2

## Data Availability

The data generated and analysed in this study are available from the corresponding author upon request.
