# Supplementary material for: Temporal differentiation of bovine airway epithelial cells grown at an air-liquid interface
Source: Sci Rep. 2018 Oct 5;8:14893. doi: 10.1038/s41598-018-33180-w (PMC6173764; doi:10.1038/s41598-018-33180-w)
Supplement: Supplementary file 1 — Supplementary information [file 41598_2018_33180_MOESM1_ESM.pdf]

Temporal differentiation of bovine airway epithelial cells grown at an air-liquid interface

Daniel Cozens[1], Erin Sutherland[1], Francesco Marchesi[2], Geraldine Taylor[3], Catherine C. Berry[4] & Robert L. Davies[1*]

[1] *Institute of Infection, Immunity and Inflammation, College of Medical, Veterinary and Life Sciences, University of Glasgow, Glasgow, UK*

[2] *School of Veterinary Medicine, University of Glasgow, Glasgow, UK*

[3] *The Pirbright Institute, Pirbright, Surrey, UK*

[4] *Institute of Molecular, Cell and Systems Biology, College of Medical, Veterinary and Life Sciences, University of Glasgow, Glasgow, UK*

*Corresponding Author

Email: robert.davies@glasgow.ac.uk

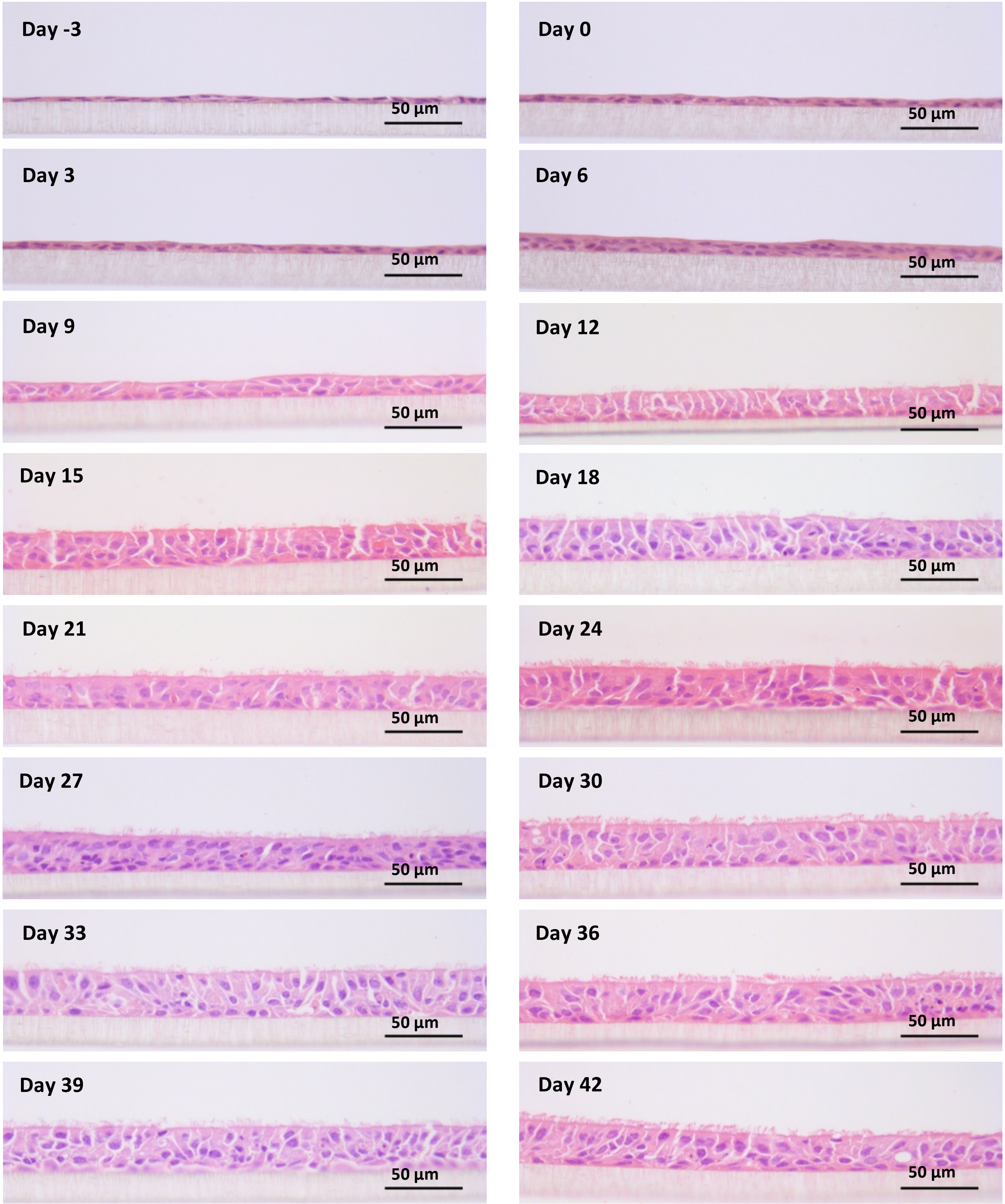

Supplementary Figure S1. Histological assessment of BBEC differentiation over time. Bronchial epithelial cell cultures were grown for the indicated number of days at an ALI (relative to the establishment of the ALI), fixed and paraffin-embedded using standard histological techniques. Sections were cut, deparaffinised and stained using H&E.

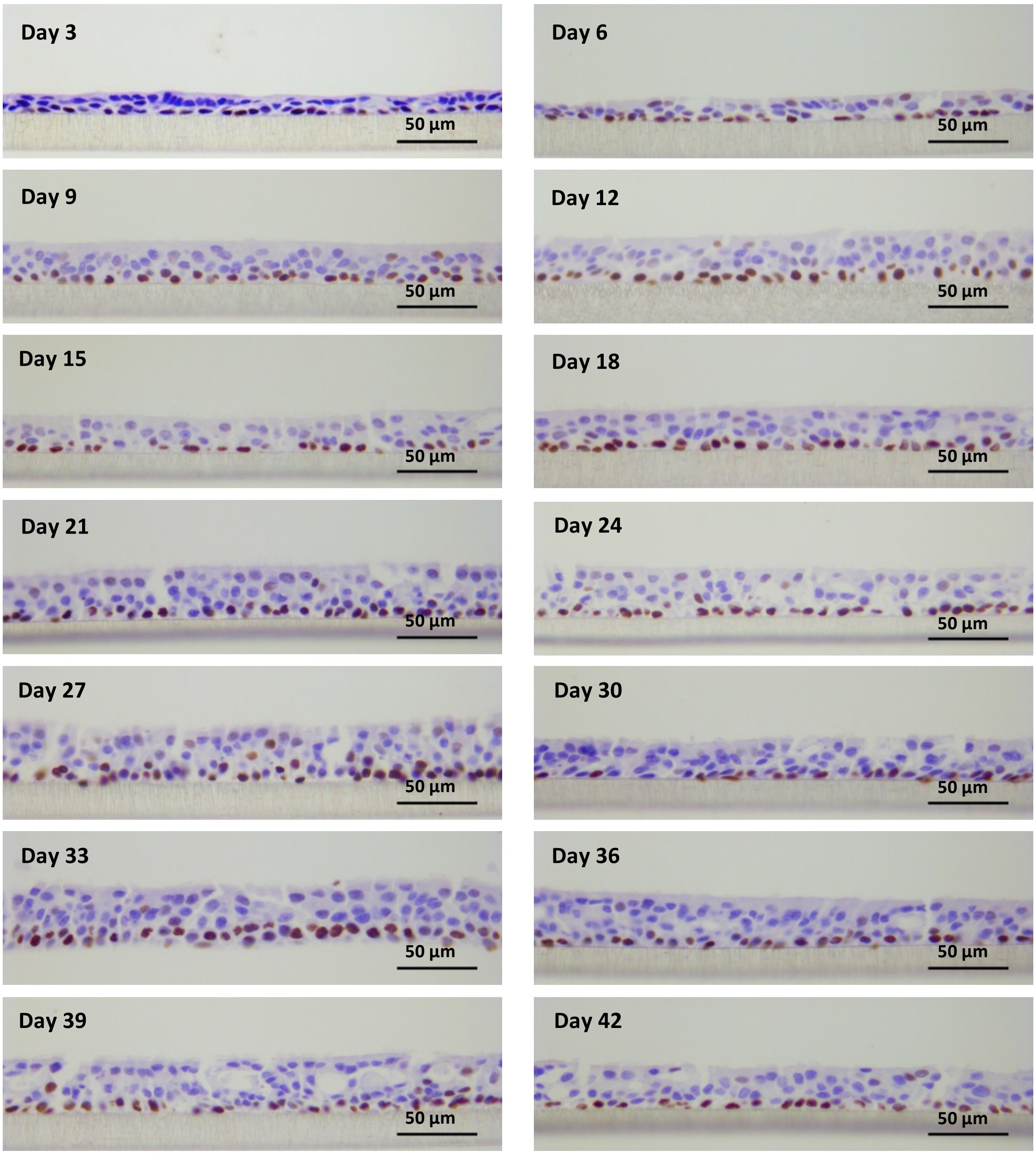

Supplementary Figure S2. Immunohistochemical assessment of basal cell distribution in BBEC cultures over time. Bronchial epithelial cell cultures were grown for the indicated number of days at an ALI (relative to the establishment of the ALI), fixed and paraffin-embedded using standard histological techniques. Sections were cut, deparaffinised, stained using immunohistochemical-labelling of basal cells (p63-labelled cells display brown nuclei) and counter-stained with Gill's haematoxylin. Note: day -3 and 0 epithelial layers were too thin to section following antigen retrieval.

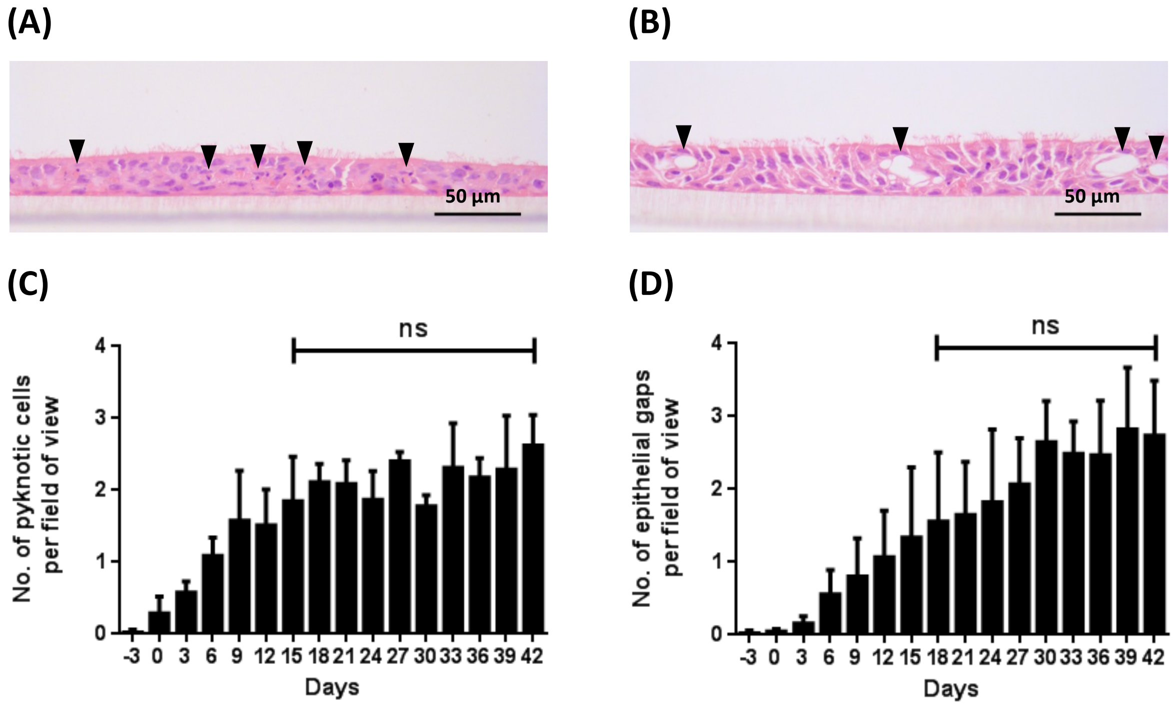

Supplementary Figure S3. Histological assessment of de-differentiation and deterioration of BBEC cultures over time. Bronchial epithelial cultures were grown for up to 42 days at an ALI, and samples were fixed at three-day intervals (from day -3 to day 42 post-ALI) and paraffin-embedded using standard histological techniques (see Fig S1). Sections were cut, deparaffinised and stained using H&E. Representative images are shown of (A) pyknotic cells (arrowheads) and (B) epithelial vacuoles/gaps (arrowheads) in BBEC cultures at days 27 and 33 post-ALI, respectively. Quantitative analysis (using ImageJ) of histological sections of BBEC layers fixed at three-day intervals (ranging from day -3 to day 42 post-ALI) was performed to assess (C) the number of pyknotic cells and (D) vacuoles/gaps per field of view. For each insert, the numbers of pyknotic cells and vacuoles/gaps were counted in each of five randomised 400x fields of view evenly distributed across the sample; three inserts were analysed at each time point and the data represents the mean +/- standard deviation from tissue derived from three different animals (n = 9). Statistical significance was tested using an Ordinary one-way ANOVA: ns = not significant.

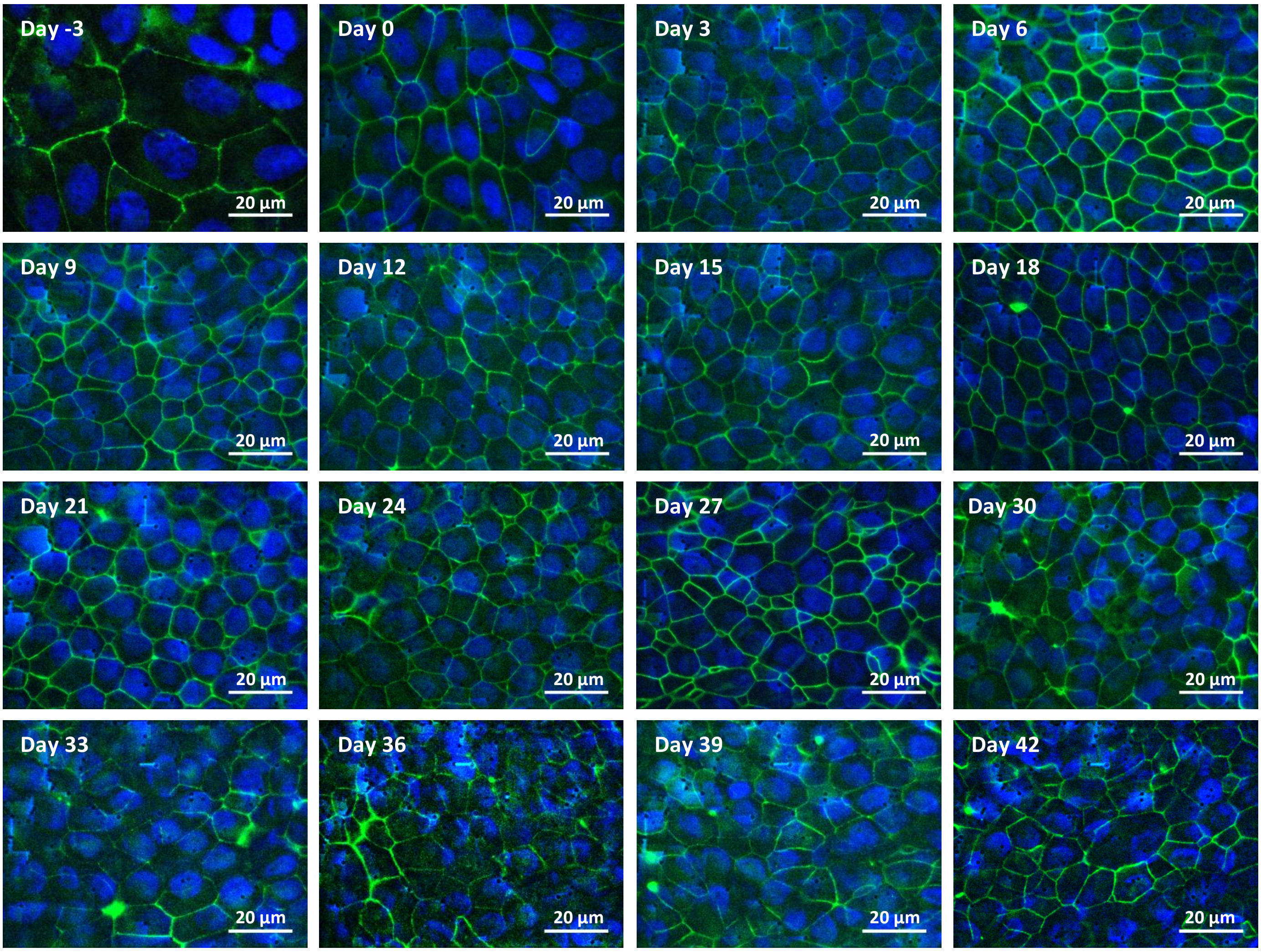

Supplementary Figure S4. Assessment of tight junction formation during differentiation of BBEC cultures over time by immunofluorescent microscopy. Bronchial epithelial cell cultures were grown for the indicated number of days at an ALI (relative to the establishment of the ALI) before fixation and immunofluorescent labelling (tight junctions - green; nuclei - blue).

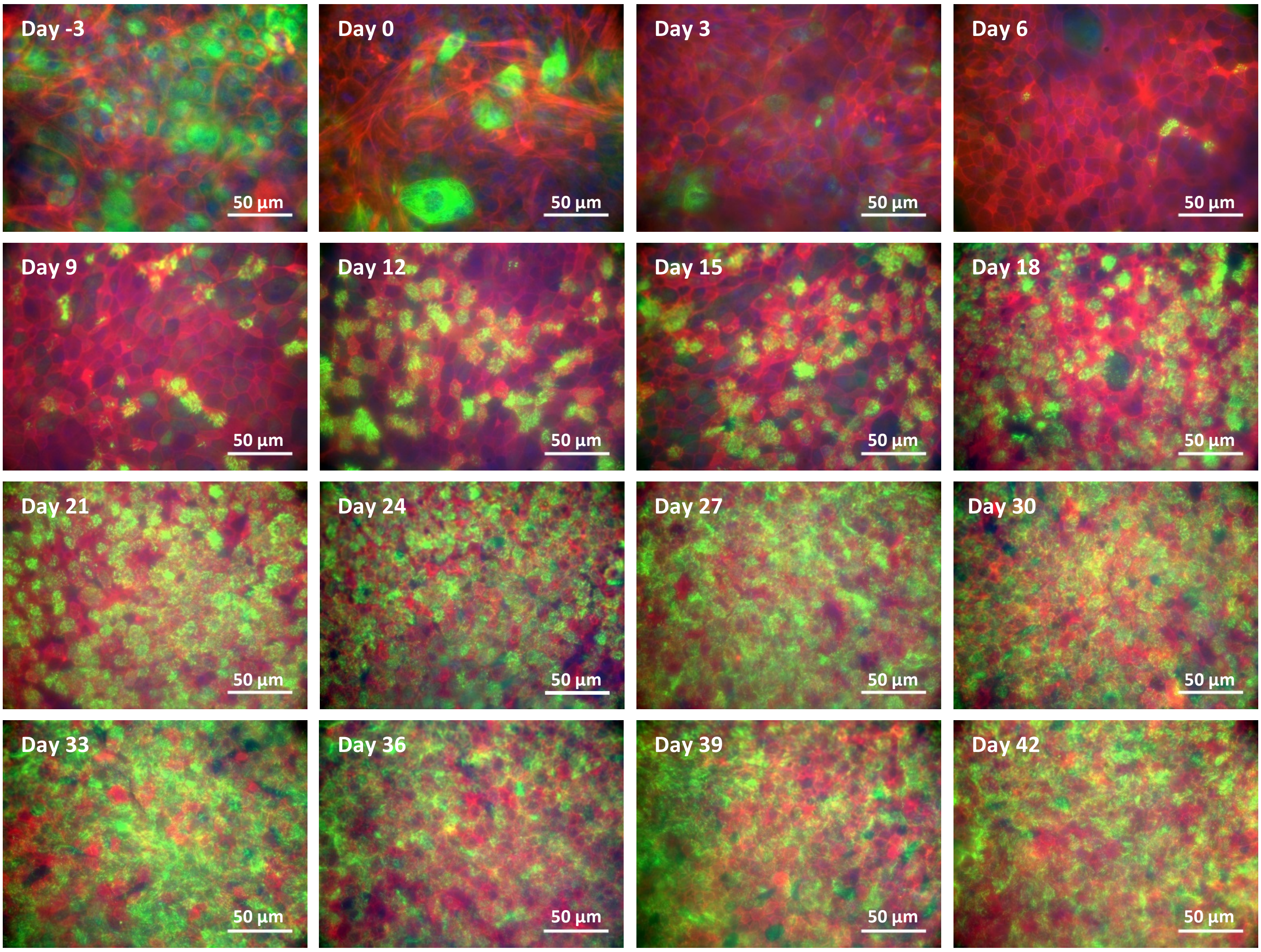

Supplementary Figure S5. Assessment of ciliogenesis during differentiation of BBEC cultures over time by immunofluorescent microscopy. Bronchial epithelial cell cultures were grown for the indicated number of days at an ALI (relative to the establishment of the ALI) before fixation and immunofluorescent labelling (cilia - green; F-actin - red; nuclei - blue).

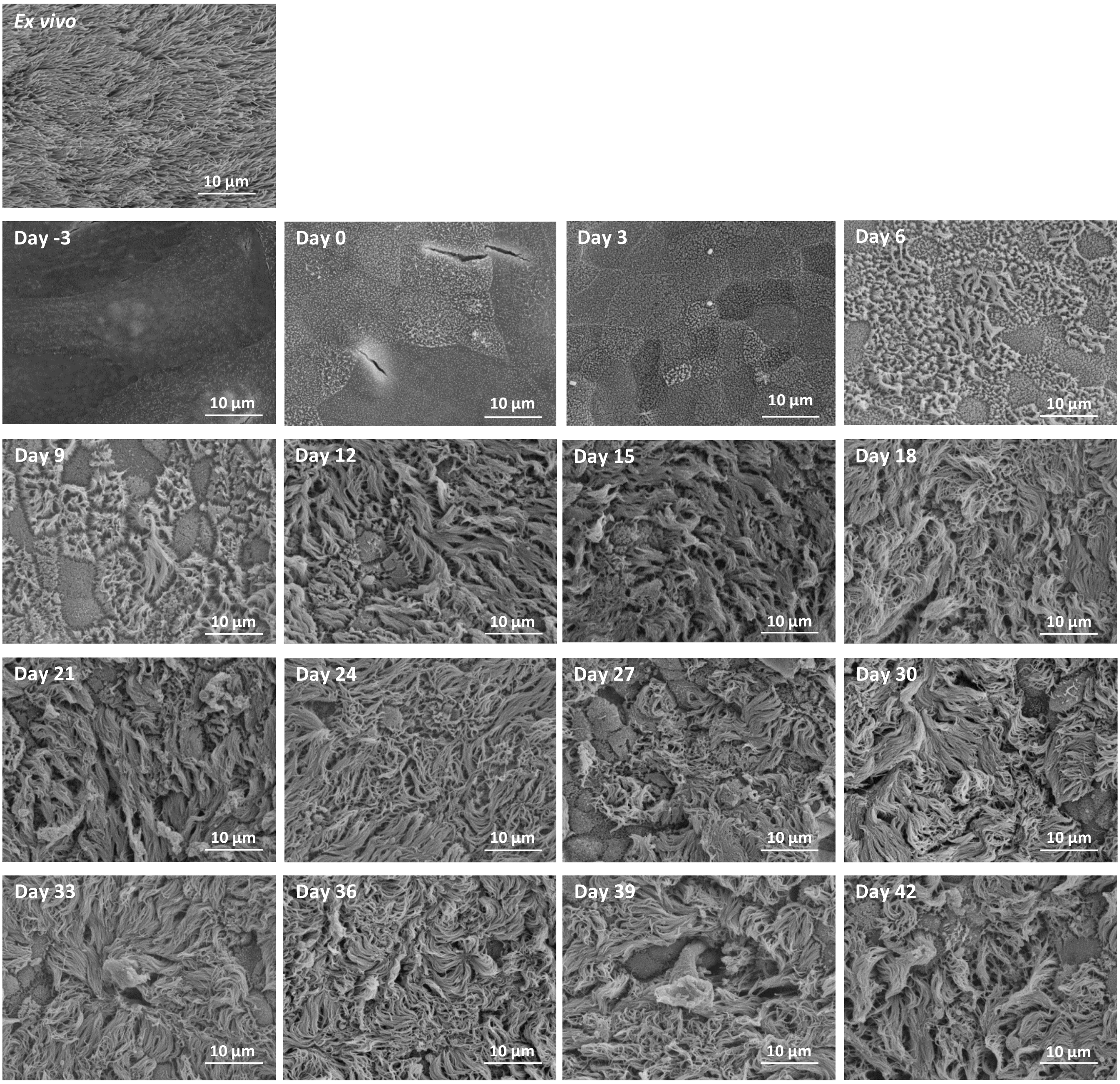

Supplementary Figure S6. Assessment of ciliogenesis during differentiation of BBEC cultures over time by SEM. Bronchial epithelial cell cultures were grown for the indicated number of days at an ALI (relative to the establishment of the ALI) before fixation and processing for SEM. *Ex vivo* bronchial epithelial tissue was dissected prior to cell extraction and similarly fixed and processed for SEM.

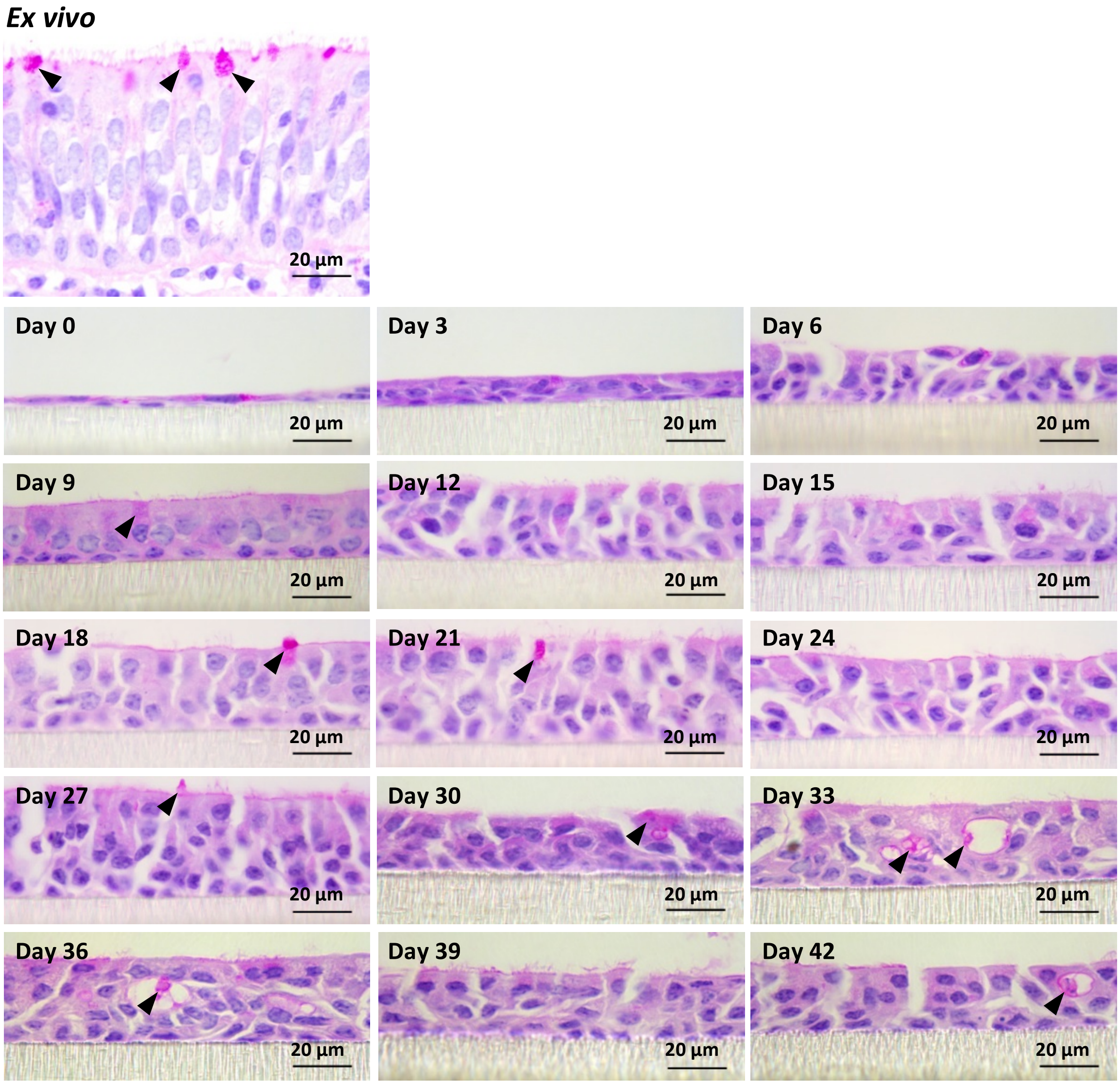

Supplementary Figure S7. Histological assessment of goblet cell formation and mucus production during differentiation of BBEC cultures over time. Bronchial epithelial cell cultures were grown for the indicated number of days at an ALI (relative to the establishment of the ALI), fixed and paraffin-embedded using standard histological techniques. Sections were cut, deparaffinised and stained with PAS. *Ex vivo* bronchial epithelial tissue was dissected prior to cell extraction and similarly fixed, sectioned and stained. PAS-positive stained goblet cells and mucus are indicated by arrowheads. At later time-points (days 33 to 42), mucus is noted in association with epithelial vacuoles/gaps.

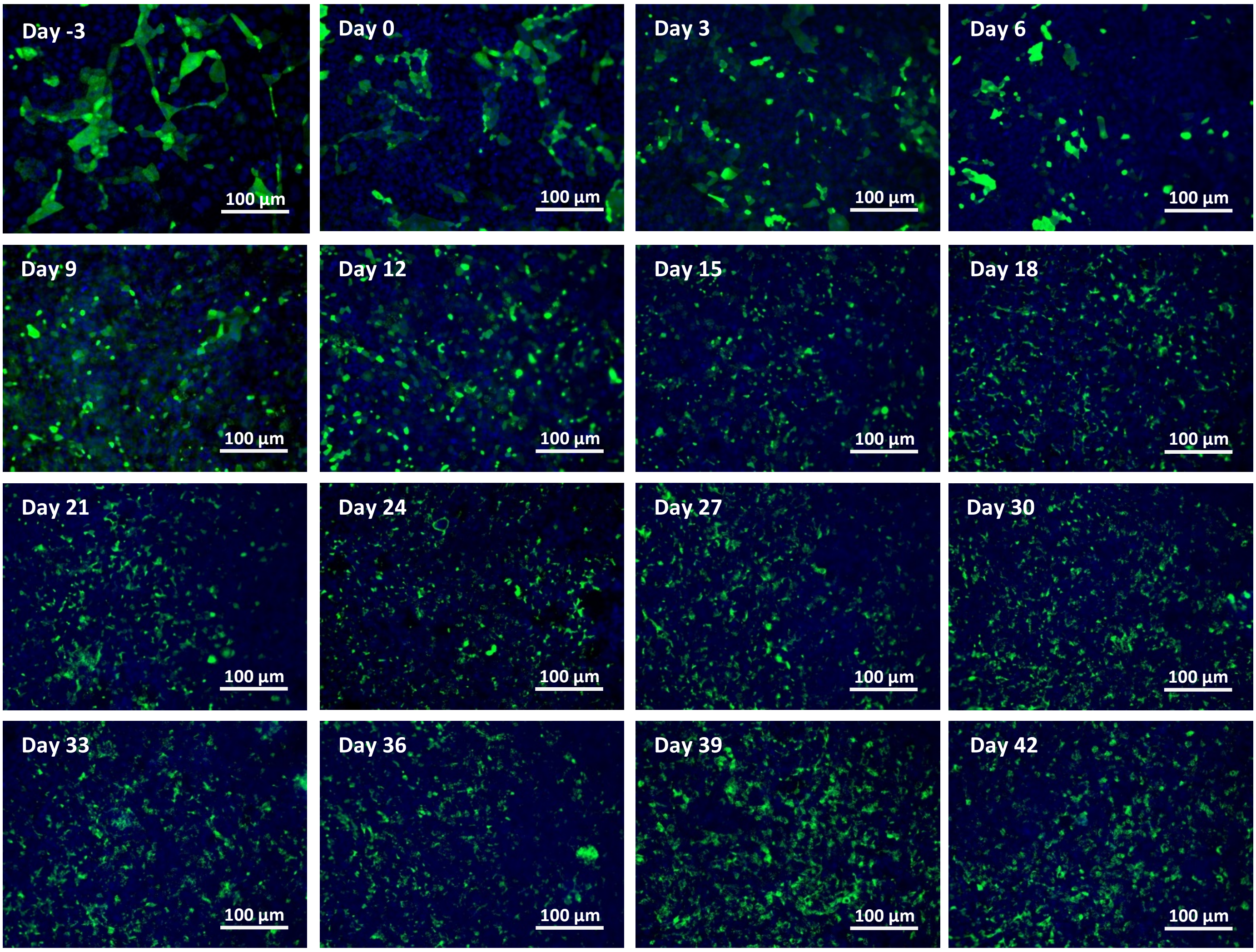

Supplementary Figure S8. Assessment of goblet cell formation and mucus production during differentiation of BBEC cultures over time by immunofluorescence microscopy. Bronchial epithelial cell cultures were grown for the indicated number of days at an ALI before fixation and immunofluorescent labelling (goblet cells/mucus - green; nuclei - blue).

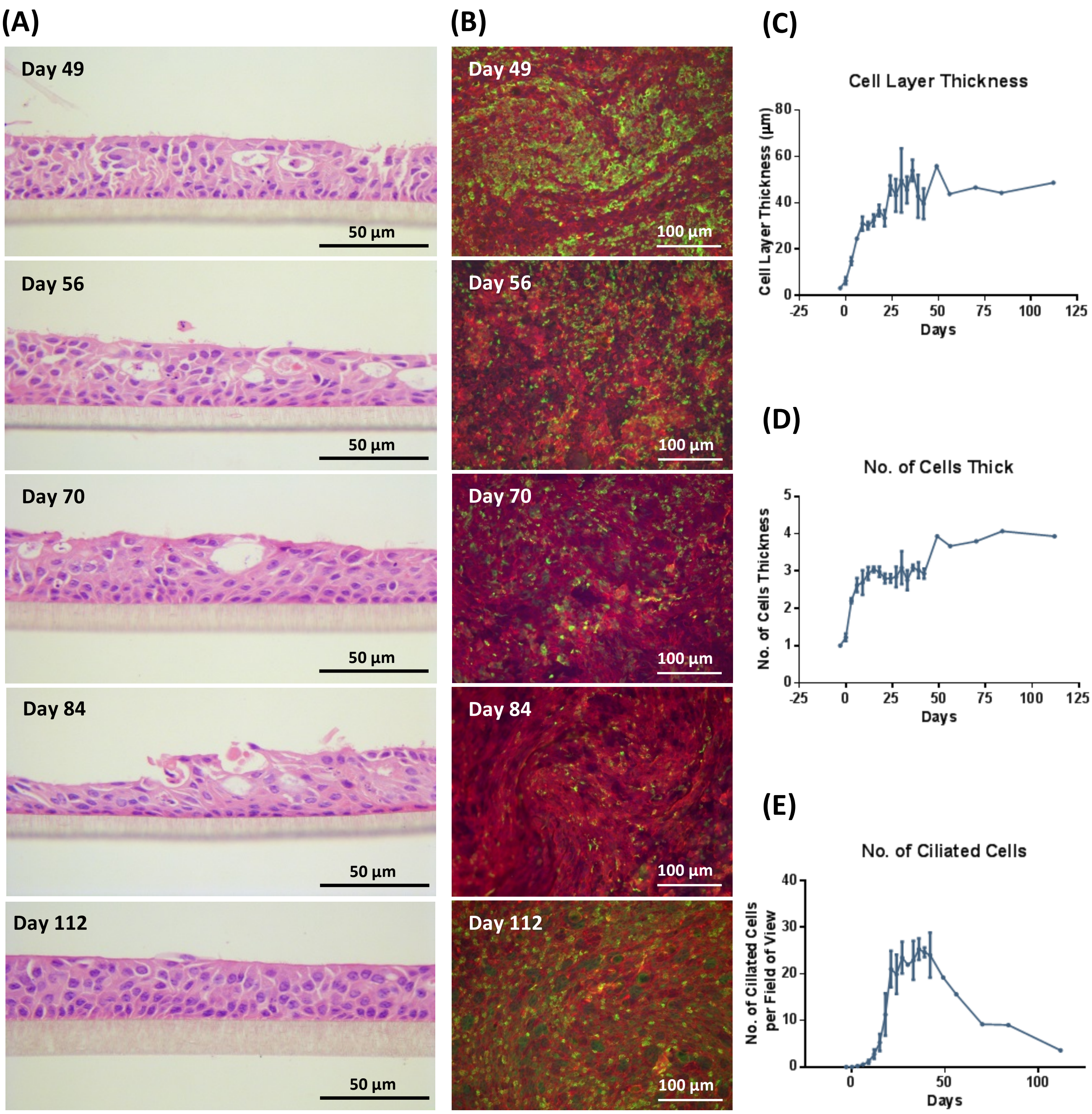

Supplementary Figure S9. Assessment of differentiated BBEC cultures maintained for 112 days post-ALI. Bronchial epithelial cell cultures were grown for the indicated number of days at an ALI (relative to the establishment of the ALI) and fixed. In (A), samples were paraffin embedded using standard histological techniques, and sections cut, deparaffinised and stained using H&E. In (B), samples were stained for immunofluorescence microscopy to allow assessment of ciliation (cilia - green; F-actin - red; nuclei - blue). Quantitative analysis (using ImageJ) was performed of histological sections of BBEC layers to assess (C) epithelial thickness, (D) the number of cell layers comprising the epithelium, and (E) the number of ciliated cells. In (C) and (D), three measurements were taken (left, centre and right) in each of five randomised 400x fields of view evenly distributed across the sample; in (E), ciliated cells were counted in each of five randomised 400x fields of view evenly distributed across the sample. Data represents the mean +/- standard deviation for tissue derived from three inserts representing a single animal (n = 3).

Supplementary Movie S1. Apicolateral arrangement of tight junctions in BBEC cultures demonstrated by confocal microscopy. Bronchial epithelial cell cultures were grown until day 21 post-ALI before fixation and immunofluorescent labelling (tight junctions - green; nuclei - blue). Image represents a 3-dimensional Z-stack model constructed using ImageJ.

Supplementary Movie S2. Differentiated BBEC cultures displaying actively-beating cilia propelling microspheres. Bronchial epithelial cells were grown until day 21 post-ALI and the movement of blue-dyed polystyrene 3 μm-diameter microspheres (Polybead; Polyscience, Inc.) was captured using a Leica Dmi1 inverted microscope.
